# The jacktree genome and population genomics provides insights for the mechanisms of the germination obstacle and the conservation of endangered ornamental plants

**DOI:** 10.1093/hr/uhae166

**Published:** 2024-06-18

**Authors:** Sheng Zhu, Xue-Fen Wei, Yu-Xin Lu, Dao-Wu Zhang, Ze-Fu Wang, Jing Ge, Sheng-Lian Li, Yan-Feng Song, Yong Yang, Xian-Gui Yi, Min Zhang, Jia-Yu Xue, Yi-Fan Duan

**Affiliations:** Co-innovation Center for Sustainable Forestry in Southern China, College of Life Sciences, Nanjing Forestry University, Nanjing 210037, China; College of Horticulture, Bioinformatics Center, Academy for Advanced Interdisciplinary Studies, Nanjing Agricultural University, Nanjing 210095, China; Co-innovation Center for Sustainable Forestry in Southern China, College of Life Sciences, Nanjing Forestry University, Nanjing 210037, China; Co-innovation Center for Sustainable Forestry in Southern China, College of Life Sciences, Nanjing Forestry University, Nanjing 210037, China; College of Ecology and Environment, Nanjing Forestry University, Nanjing 210037, China; College of Horticulture, Bioinformatics Center, Academy for Advanced Interdisciplinary Studies, Nanjing Agricultural University, Nanjing 210095, China; Co-innovation Center for Sustainable Forestry in Southern China, College of Life Sciences, Nanjing Forestry University, Nanjing 210037, China; Co-innovation Center for Sustainable Forestry in Southern China, College of Life Sciences, Nanjing Forestry University, Nanjing 210037, China; Co-innovation Center for Sustainable Forestry in Southern China, College of Life Sciences, Nanjing Forestry University, Nanjing 210037, China; Co-innovation Center for Sustainable Forestry in Southern China, College of Life Sciences, Nanjing Forestry University, Nanjing 210037, China; Co-innovation Center for Sustainable Forestry in Southern China, College of Life Sciences, Nanjing Forestry University, Nanjing 210037, China; College of Horticulture, Bioinformatics Center, Academy for Advanced Interdisciplinary Studies, Nanjing Agricultural University, Nanjing 210095, China; Co-innovation Center for Sustainable Forestry in Southern China, College of Life Sciences, Nanjing Forestry University, Nanjing 210037, China

## Abstract

*Sinojackia* Hu represents the first woody genus described by Chinese botanists, with all species classified as endangered ornamental plants endemic to China. Their characteristic spindle-shaped fruits confer high ornamental value to the plants, making them favored in gardens and parks. Nevertheless, the fruits likely pose a germination obstacle, contributing to the endangered status of this lineage. Here we report the chromosome-scale genome of *S. xylocarpa*, and explore the mechanisms underlying its endangered status, as well as its population dynamics throughout evolution. Population genomic analysis has indicated that *S. xylocarpa* experienced a bottleneck effect following the recent glacial period, leading to a continuous population reduction. Examination of the pericarp composition across six stages of fruit development revealed a consistent increase in the accumulation of lignin and fiber content, responsible for the sturdiness of mature fruits’ pericarps. At molecular level, enhanced gene expression in the biosynthesis of lignin, cellulose and hemicellulose was detected in pericarps. Therefore, we conclude that the highly lignified and fibrotic pericarps of *S. xylocarpa*, which inhibit its seed germination, should be its threatening mechanism, thus proposing corresponding strategies for improved conservation and restoration. This study serves as a seminal contribution to conservation biology, offering valuable insights for the study of other endangered ornamental plants.

## Introduction

*Sinojackia* Hu (Ericales: Styracaceae) represents the first woody genus discovered and described by Chinese plant taxonomists [1]. It is a rare Chinese endemic genus comprising merely seven species: *S. henryi* (Dummer) Merr., *S. huangmeiensis* J. W. Ge & X. H. Yao, *S. microcarpa* Tao Chen & G. Y. Li, *S. oblongicarpa* Tao Chen & T. R. Cao, *S. rehderiana* Hu, *S. sarcocarpa* L. Q. Luo and *S. xylocarpa* Hu [2]. All seven species are listed as a Grade II endangered protected plant in China, mainly due to their small population size and rare, scattered distributions (http://www.iplant.cn/rep/protlist).

**Figure 1:**
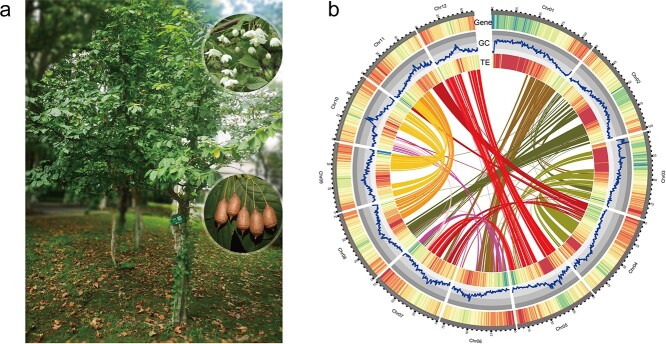
Morphology and high-quality genome assembly of the *Sinojackia xylocarpa*. **a** Flowering and fruiting branches of *S. xylocarpa*. **b** Genome features across 12 chromosomes of *S. xylocarpa*. From the outermost to innermost circles are chromosome ideograms, gene density (from blue to red), GC content, TE (transposable elements) density (from blue to red), and collinear genomic blocks.

*S. xylocarpa* Hu is the representative and first-discovered species in the genus, known as the ‘jacktree’ in honor of the botanist John George Jack [1]. It is a flowering deciduous shrub or dwarf tree, reaching heights of about 4.6~6.1 metres and a diameter of nearly 10 centimetres at breast height. *S. xylocarpa* has a highly aesthetic and ornamental value, primarily due to its egg-shaped woody fruits (drupes) that resemble a balanced weight set (‘Chengtuo’ in Chinese). In autumn, the abundant egg-shaped fruits hang in cascading clusters, adding to its distinctive ornamental value, which can enhance the beauty of courtyards or parks. Additionally, during the spring flowering period, the tree is covered with abundant pure white blossoms (Fig. 1a). However, the extant population size of *S. xylocarpa* is very limited in nature, and its distribution is also extremely fragmented in the subtropical zone of Eastern China (e.g., Jiangsu and Zhejiang Provinces). Its small population size and narrow distribution are mainly attributed to its low germination rate, probably caused by the recalcitrant seeds [3]. Therefore, *S. xylocarpa* is classified as vulnerable (VU) in the International Union for Conservation of Nature and Natural Resources (IUCN) Red List of Threatened Species (https://www.iucnredlist.org/), attempting to raise public attention and promote protection. Botanical gardens, the primary protectors of endangered plants, have taken prioritizing action, introducing wild *S. xylocarpa* individuals into botanical gardens in Eastern China, such as Nanjing Botanical Garden Mem. Sun Yat-Sen and Shanghai Chenshan Botanical Garden, for decades.

Such strategies can certainly benefit the conservation of *S. xylocarpa* to a certain extent, potentially saving the species from extinction. In other words, by understanding the mechanism underlying the poor germination rate of *S. xylocarpa* seeds, we may be able to propose a highly targeted and efficient conservation plan to facilitate its sustainable recovery. Anatomically, *S. xylocarpa* seeds have extremely rigid external structure, consisting of indehiscent exocarp, corky mesocarp, lignified endocarp, and hard seed coat. The tough outer structure not only protects the seeds of *S. xylocarpa* but also renders them difficult to germinate within 2 to 3 years (http://www.efloras.org/). Unfortunately, the molecular mechanism underlying the mechanical barriers of the fruit pericarp to germination remains largely unknown, mainly due to the absence of a high-quality nuclear genome assembly for *S. xylocarpa*.

The reference genome is a fundamental resource for assessing, protecting, and restoring the biodiversity of endangered plants [4], including *Ostrya rehderiana* [5], *Davidia involucrata* [6], and *Acer yangbiense* [7, 8]. Genomic comparative analysis of the endangered ironwood *O. rehderiana* and its non-endangered relative *Ostrya chinensis* revealed the genomic effects of population collapse in this species [5]. Genomic analysis of the endangered dove-tree *D. involucrata* demonstrated that its endangered status might be primarily influenced by genomic factors, genetic diversity and population structure [6]. Whole-genome resequencing of 105 individuals from ten extant *A. yangbiense* populations revealed that their small population size might be related to low genetic diversity, repeated bottleneck events, and deleterious mutation load [8]. Genetic diversity of endangered plant species is crucial for conserving their genetic resources [9]. At present, our knowledge of the phylogeny and genetic diversity of *Sinojackia* and Styracaceae is limited to chloroplast genomes [10–14] and microsatellite markers [15–17].

Many plants in the order Ericales possess high economic and ornamental values, for instance, *Rhododendron simsii* (azalea) [18], *Actinidia eriantha* (kiwifruit) [19], *Diospyros oleifera* (persimmon) [20] and *Camellia sinensis* (tea) [21]. As a result, the genomes of these valuable plants have already been sequenced; however, no genomes of Styracaceae species are available. Here, we reported a chromosome-level assembly of the *S. xylocarpa* genome, which is the first sequenced nuclear genome in Styracaceae. Based on the high-quality reference genome, we conducted comprehensive analyses combining bioinformatics and experiments. Our study provides evidence from anatomy, gene expression, and population genomics to explore the mechanisms for the germination barriers of *S. xylocarpa* seeds and its narrow distribution. This work establishes a genomic foundation for investigating the molecular biology and evolution of *S. xylocarpa,* as well as for its biodiversity maintenance and restoration.

## Results

### Sequencing, assembly, and annotation of the *S. xylocarpa* genome

The genomic survey for the *S. xylocarpa* was conducted using Illumina data, and the estimated results by *k-mer* analysis (*k-mer* = 19) suggests a genome size of approximately 1.01 Gb, with a heterozygosity of around 0.91% (Fig. S1, see online supplementary material). To obtain a high-quality genome of *S. xylocarpa*, we employed a sequencing strategy combining high-fidelity (HiFi) long reads with a depth of nearly 17× (16.97 Gb) and Hi-C data of approximately 91× depth (98.6 Gb). The HiFi long reads were initially *de novo* assembled into 2138 contigs with a total length of 1072 Mb (N50 = 1.5 Mb). Subsequently, these contigs were ordered and oriented into 1168 scaffolds (N50 = 78.8 Mb) using Hi-C data. In total, 982 contigs (986 Mb) were anchored to 12 pseudochromosomes, representing for 91.98% of the entire *S. xylocarpa* genome (Fig. S2, see online supplementary material). Therefore, the resulting *S. xylocarpa* assembly is consistent with the estimated genome and should have achieved the chromosome-level in terms of continuity (Fig. 1b and Table 1).

**Table 1:** Statistics of the *S. xylocarpa* genome assembly and annotation

**Statistic**	**Size**
Genome size (Gb)	1.072
Number of scaffolds	1168
Scaffold length (bp)	1072,438,512
Scaffold N50 size (bp)	78 843 817
Scaffold N90 size (bp)	61 418 785
Number of contigs	2138
Contigs length (bp)	1072,341,512
Contigs N50 size (bp)	1 500 000
Contigs N90 size (bp)	269 899
Number of genes	40 924
Tandem repeats rate (%)	6.98
Transposable elements rate (%)	53.75
Complete BUSCO score (%)	94.86
GC (%)	39.25

Gene annotation for the *S. xylocarpa* genome predicted a total of 40 924 protein-coding gene models using three gene prediction approaches: transcriptome-based, homology-based, and *ab initio* prediction. Among the predicted genes, 26 707 (65.26%) supported by transcriptome data, and 39 703 genes (97.02%) could be functionally annotated (Table S1, see online supplementary material). Our annotation identifies 1531 (94.86%) of the embryophyta benchmarking universal single-copy orthologs (BUSCOs) in the *S. xylocarpa* genome, with 1368 (84.76%) in single-copy and 163 (10.10%) in duplicate, indicating a high quality of gene prediction of *S. xylocarpa* genome.

Repeat sequences were also annotated, comprising 53.75% (576 Mb) of the assembly. Retroelements are the most abundant components (45.13%) among the repetitive sequences, with *Gypsy* and *Copia* comprising 21.39% and 8.46% of the genome (Table S2, see online supplementary material), respectively. Additionally, we identified 2111 transfer RNAs, 5315 ribosomal RNAs, 168 microRNAs, 152 small nuclear RNAs, and 129 small nucleolar RNAs in the *S. xylocarpa* genome.

### Phylogenetic position of Ericales and relationships of lineages within Ericales

**Figure 2:**
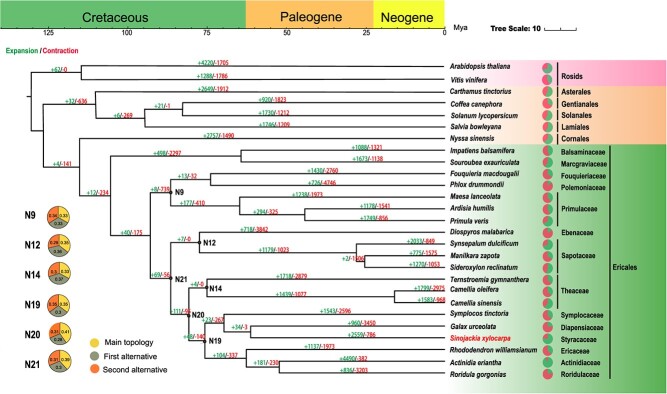
Genome evolution analysis of *Sinojackia xylocarpa*. Expansion and contraction of gene families and phylogenetic relationships and divergence times between *S. xylocarpa* and other plant species. The light green numbers represent the numbers of expanded gene families, and the red numbers represent the numbers of contracted gene families.

A total of 1291 low-copy orthologous genes were extracted from 27 angiosperm genomes and used to construct the sequence dataset for phylogenetic analyses. The concatenated amino acid dataset supported Ericales as the sister group of Cornales, another basal asterid lineage, with strong bootstrap support (100%, Fig. 2). The result based on the coalescent method was congruent (Fig. S3, see online supplementary material), suggesting a robust sister relationship for the two basal asterid orders.

*S. xylocarpa* was resolved as the sister to *Galax urceolata*, a species in the Diapensiaceae family, and the two corresponding taxa diverged approximately 61.18 million years ago (Mya). The two species were sisters to *Symplocos tinctoria* (Symplocaceae) (Fig. 2). The monophyly of these three families has been robust and consistent across phylogenetic inferences based on organellar and nuclear data [22, 23]. This monophyletic group was further recovered as the sister to another lineage comprising Roridulaceae, Actinidiaceae, and Ericaceae. However, the relationships between some other lineages in Ericales remain ambiguous due to an ancient rapid radiation [23], with multiple lineages collapsed into a polytomy [24], including Theaceae, Sapotaceae, Ebenaceae, and Primulaceae. Our concatenated super-matrix provided hypotheses for these unresolved lineages, with Theaceae being sister to Pentaphylacaceae, Sapotaceae being sister to Ebenaceae, and Primulaceae being sister to the Polemoniaceae-Fouquieriaceae lineage. However, these inferences were all weakly supported and sometimes conflicted with the results from the coalescent analyses (Fig. S3, see online supplementary material). Incongruence and/or weak support in phylogenetic reconstruction may be due to incomplete lineage sorting (ILS), a consequence of random distribution of ancestral allelic polymorphism in derived lineages through rapid radiation [25]. Therefore, we examined the single gene trees regarding the relationships of these unresolved lineages. As expected, none of the relationships were predominant among all single gene trees, with nearly equal support for alternative relationships (Fig. 2; Fig. S3 , S4, and Table S3, see online supplementary material), suggesting substantial likely ILS during the evolution of Ericales.

### Whole-genome duplication (WGD) events and karyotype evolution of *S. xylocarpa*

**Figure 3:**
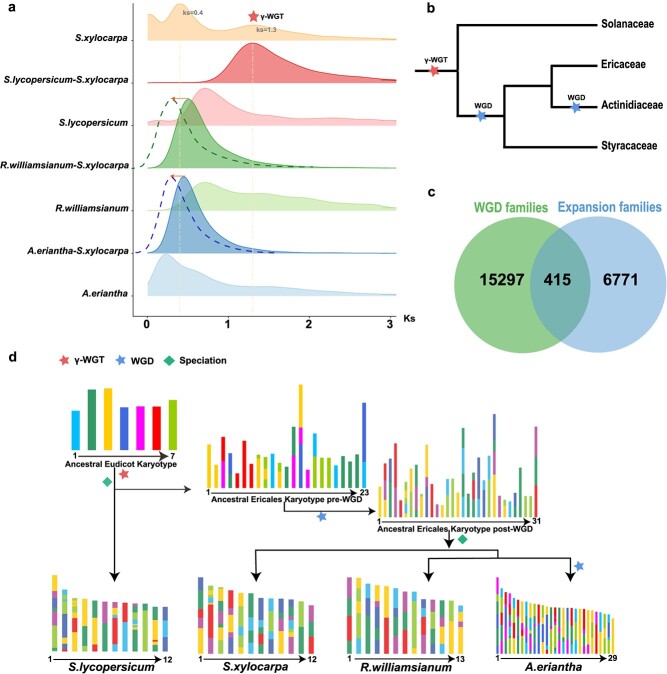
Whole-genome duplication in the *Sinojackia xylocarpa* genome. **a** Distribution of overall synonymous substitution levels (Ks) for paralogs found in syntenic blocks of *Actinidia eriantha*, *Rhododendron williamsianum*, *S. xylocarpa*, and *Solanum lycopersicum* and for orthologs between *R. williamsianum* and *S. xylocarpa* and between *A. eriantha* and *S. lycopersicum*. The yellow dotted line indicates two WGD events in *S. xylocarpa*. The Ks distribution of *S. xylocarpa* showed two peaks, one at approximately 0.3 (WGD 2) and another at approximately 1.3 (WGD 1). The arrows in different colors indicate overestimations (to the right) of the divergence events and point to the Ks values after corrections of different substitution rates based on that in *S. xylocarpa*. The dotted curves also show the orthologous distributions after substitution rate corrections. **b** Genome duplication in Ericales and outgroup. A red star indicates the core-eudicot whole-genome triplication (γ-WGT) event that occurs in conjunction with other species. Two blue stars indicate a WGD event that is commonly experienced in Ericales and an Actinidiaceae-specific WGD, respectively. **c** The Venn diagram shows the relationships among WGD families and expanded families in the *S. xylocarpa* genome. **d** Chromosome karyotype evolution from AEK to *S. xylocarpa*. A red star indicates a WGT event. A blue star indicates a WGD event. Green rhombus indicates speciation event. Different color blocks represent different ancestral chromosomes.

Previous genomic studies identified several independent whole-genome duplications (WGDs) in different Ericales taxa [18, 26, 27]. By comparing collinear genomic blocks (Fig. S5, see online supplementary material) and calculating the synonymous substitution rate (*K*_S_) of all paralogs in collinear regions (anchored paralogs) in the *S. xylocarpa* genome, we identified two *Ks* signature peaks approximately 1.3 and 0.4 (Fig. 3a), respectively, suggesting two rounds of WGD events.

To accurately date the two WGDs occurred in *S. xylocarpa,* we compared the orthologous *Ks* distributions of *S. xylocarpa* with that of tomato (*Solanum lycopersicum*), azaleas (*Rhododendron williamsianum*) and kiwifruit (*A. eriantha*) (Fig. 3a). The *Ks* peak value of 1.3 for *S. xylocarpa* should correspond to the whole-genome triplication (WGT, γ) shared by all eudicots [27]. Therefore, it is logical that the peak of γ-WGT is slightly larger than the peak representing the divergence between *S. xylocarpa* and tomato. The more recent polyploidy event identified in *S. xylocarpa* should be a WGD, as indicated by the 2:1 ratio of collinear blocks (Fig. S5 and S6, see online supplementary material). However, the differential *Ks* among *S. xylocarpa*, azaleas, and kiwifruit resulted in an overestimate for the divergences between *S. xylocarpa* and the other two Ericlaes taxa. We therefore conducted a relative rate test to adjust the synonymous substitution, thus recognizing the more recent WGD in *S. xylocarpa* to be likely shared by Styracaceae, Diapensiaceae, and Symplocaceae (Fig. 3b).

When examining the retained paralogous genes (15712) after the recent WGD in *S. xylocarpa*, we found that they are partially overlapping with the expanded gene families (2559 families, 7186 genes, Fig. 3c) since the divergence with *G. urceolata*. GO analysis of these retained paralogs indicated that they are mainly enriched in ‘response to chemical and organic substance’, ‘developmental, catabolic and biological process’, and ‘organic substance catabolic and organonitrogen compound metabolic process’ with respect to ‘biological progress’, ‘ligase, transporter, acyltransferase, and kinase activity’, and ‘ATP binding’ with respect to ‘molecular function’ (Fig. S7, see online supplementary material).

WGDs are important evolutionary events that profoundly influence the genome evolution of organisms. Therefore, we reconstructed the process of karyotype variation of *S. xylocarpa* from the common ancestor of eudicots (Fig. 3d). The ancestral eudicot karyotype (AEK) was inferred to possess seven ancestral chromosomes before the γ-WGT [28]. Afterward, the triplicated AEK (21 chromosomes) underwent at least 10 chromosome fusions, 18 breaks, and six losses to form the karyotype in the common ancestor of *S. xylocarpa*, azaleas, and kiwifruit before their shared WGD (23 chromosomes). The recent WGD doubled the chromosome number, and 15 fusions, 0 breaks, and 0 losses reduced the chromosome number to 31 in the ancestor. Finally, extensive karyotype variations occurred (19 fusions, 0 breaks and 0 losses), leading to the current karyotype in *S. xylocarpa*.

### Population genetic analyses of *S. Xylocarpa*

**Figure 4:**
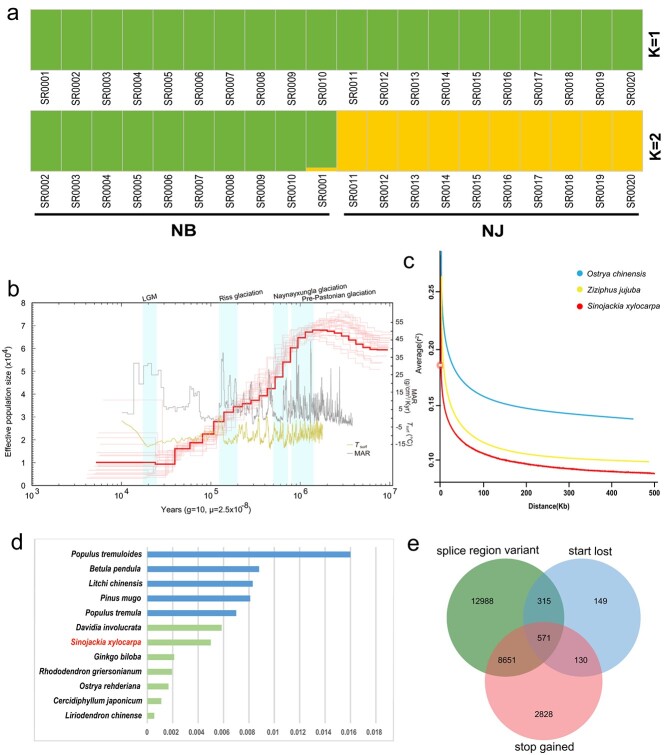
Genetic diversity and demographic history of *Sinojackia xylocarpa*. **a** Admixture analysis with the number of clusters (K) ranging from 1 to 2. **b** Demographic history of *S. xylocarpa*. The last glacial maximum (LGM), Riss glaciation, the Naynayxungla glaciation, and Pre-Pastonian glaciation are highlighted in light-blue vertical bars. **c** Linkage disequilibrium (LD) decay of the *S. xylocarpa* populations and the two non-endangered species, including *Ostrya chinensis* and *Ziziphus jujuba*. **d** The observed genetic diversity (π) of *S. xylocarpa* and the remaining 17 species. **e** The Venn diagram of genes having three detrimental mutations, including splice region variant, loss of start codon, or gain of stop codons.

Natural populations of *S. xylocarpa* were found only in Nanjing, Jiangsu Province (118.39°N, 32.05°E) and Ningbo, Zhejiang Province (121.40°N, 30.08°E). We collected ten individuals from each of the two populations (Fig. S8, see online supplementary material) and conducted whole-genome resequencing on all 20 samples and obtained raw data with an average sequencing depth of 18.6×. Using a stringent GATK pipeline and strict filtering, we obtained a total of 43 817 152 single-nucleotide polymorphisms (SNPs), with approximately 65.79% of SNPs located in the intergenic regions and the remaining approximately 34.21% found in genic regions (Table S4, see online supplementary material). Phylogenetic analysis and principal component analysis (PCA) both revealed the clear divergence between the Ningbo population (Zhejiang Province) and the Nanjing population (Jiangsu Province) (Figs. S9 and S10, see online supplementary material). Based on the results of ADMIXTURE analysis, *K* = 1 was the best-fitted model (Table S5, see online supplementary material), indicating the small effective population size (*N*_e_) usually possessed by the endangered species. However, the two populations (Nanjing and Ningbo) of *S. xylocarpa* were distinctly separated when K = 2 (Fig. 4a), consistent with the results from the PCA and the phylogenetic tree. We then estimated the historical changes of *N*_e_; all sequenced *S. xylocarpa* individuals from the two populations showed a continuous decreasing trend over the recent one million years (Fig. 4b; Fig. S10 and Table S6, see online supplementary material), revealing the experienced bottleneck effect that was likely involved in the recent glacial periods. The gene flow analysis indicated the existence of bidirectional genetic communications between the two populations in NJ and NB, as well as a more predominant flow from NB to NJ (Fig. S12 and Table S7, see online supplementary material). This indicated that before the glacial period *S. xylocarpa* might have a continuous distribution area with frequent genetic exchange between different populations. During the glacial periods, its gene flow might be gradually restricted by habitat fragmentation and anthropogenic activities [29].

*S. xylocarpa* had a relatively low genome-wide nucleotide diversity (π) value (5 × 10^−3^), similar to other endangered plants, such as *O. rehderiana* (1.66 × 10^−3^) [5], *D. involucrata* (5.85 × 10^−3^) [6], and *A. yangbiense* (3.13 × 10^−3^) [8] (Fig. 4d), suggesting common genomic characteristics among endangered plants. Theoretically, the low genomic diversity appears to be an inevitable consequence of being endangered species, due to their small population size. This was also illustrated by the slow linkage disequilibrium (LD) decay within *S. xylocarpa*, in which half of the maximum r^2^ was not attained until ~500 kb. *S. xylocarpa* showed faster LD decay than two non-endangered species, including *O. chinensis* [5] and *Ziziphus jujube* [30] (Fig. 4c). We further estimated the inbreeding level via runs of homozygosity (ROH) (Fig. S13, see online supplementary material). The short ROHs occupied the majority (>99%) of the proportion, indicating the historical population bottleneck within *S. xylocarpa*.

Finally, we identified a total of 25 653 loss-of-function variations (Fig. 4e; Table S8, see online supplementary material) in *S. xylocarpa*. Among them, the variations belonging to start lost, stop gained, and splice region variant were 1165, 12 205, and 22 542, respectively. We found that 26 genes in the lignin synthesis pathway produced deleterious variations, with the most abundant being the splice region variant (Table S9, see online supplementary material). For identification of environment-associated genetic variants, we used different climate and soil factors to explore the environmental adaptation of *S. xylocarpa*. The results revealed that six environmental factors were most correlated with its environmental adaptation, with three temperature-related factors, namely annual mean temperature, isothermality, and mean temperature of warmest quarter, and three others related to soil conditions, namely soil clay content, cation exchange capacity, and soil organic carbon content have a significant impact on the growth of *S. xylocarpa* (Fig. S14 and Table S10, see online supplementary material).

### Highly lignified pericarps leading to the germination obstacle for *S. xylocarpa*

**Figure 5:**
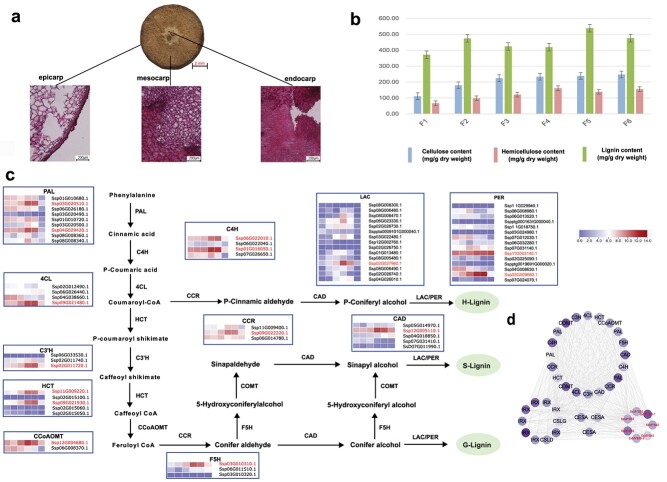
The lignin biosynthesis pathways in *Sinojackia xylocarpa* fruit pericarp. (**a**) The cross-sections of *S. xylocarpa* pericarp. Lignin distributions of epicarp, mesocarp, and endocarp (from left to right) in *S. xylocarpa* pericarp were visualized via Safranin-O staining. (**b**) Mean values of cellulose, hemicellulose, and lignin contents of *S. xylocarpa* fruits during six different developmental stages. (**c**) Genes in the biosynthesis pathway of lignin and their expressions in pericarps. The genes marked in red are named as genes in the red module of the WGCNA analysis. (**d**) Gene regulatory networks associated with cellulose, hemicellulose, and lignin synthesis in the red module by WGCNA analysis, including the R2R3-MYB gene family involved in the regulation of lignin synthesis.

The stony pericarps can impose mechanical constraints on seed germination. A previous study proposed that the hard endocarp is likely the reason that inhibits the germination of *S. xylocarpa* seeds [3], which leads to the current small population size and extremely narrow distribution. To verify this hypothesis, we conducted an anatomical and staining experiment towards the longitudinal section of *S. xylocarpa* fruits. We observed that the seeds in the center were wrapped by hard and thick fruit pericarps composed of firm woody tissues and fibres, which is obviously the result of massive lignin and cellulose deposition (Fig. 5a). We subsequently measured the content of lignin, cellulose, and hemicellulose in pericarps at different developmental stages of fruits and found that the *S. xylocarpa* pericarps have continued to accumulate lignin, cellulose, and hemicellulose during the developmental process (Fig. 5b). This accumulation is likely the reason for the solid pericarps. Regarding the molecular mechanism, since the gene copy number of the LBP in *S. xylocarpa* does not significantly outnumber other species (Table S19, see online supplementary material), the underlying mechanism is likely related to gene expressions. When examining the lignin biosynthesis pathway (LBP), it is noticeable that some genes in the LBP showed a similar increasing expression pattern (gene IDs in red, Fig. 5c;Fig. S15, see online supplementary material), which is consistent with the trend of lignin content accumulation during fruit development. These genes include two PALs (phenylalanine ammonia lyases), two C4Hs (cinnamate 4-hydroxylases), two HCTs (shikimate hydroxycinnamoyl transferases), one C3H (p-coumarate 3-hydroxylase), one 4CL (4-coumaric acid: coenzyme A ligase), one COMT (catechol-O-Methyltransferase), one F5H (ferulate-5-hydroxylase), one CCoAOMT (caffeoyl-CoA 3-O-methyltransferase), one CCR (cinnamoyl-coenzyme A reductase), and one CAD (carbamoyl-phosphate synthetase), covering every catalysing step. There are other copies of LBP genes that did not show an increasing pattern or were expressed at low levels during fruit development. For example, one SxCAD4/5 copy (Ssp12G005110.1) displayed an increased and high expression, but the other copy (Ssp05G014970.1) was lowly expressed (Fig. 5c). We infer that these copies may be responsible for lignin biosynthesis in other organs/tissues, but not in pericarps, with different organ/tissue expressing specificity, or they may simply represent functional redundancy. Additionally, all LBP genes have undergone strong purifying selection during evolution (Table S11, see online supplementary material).

Naturally, in our constructed co-expression networks, the LBP genes showing a coincident increasing pattern were classified into the same module (red) by WGCNA (Fig. 5d; Table S12, see online supplementary material), suggesting that they are all potentially associated with lignin biosynthesis in *S. xylocarpa* pericarps. The high expression of some LBP genes may be associated with their tandemly arranged physical arrays on chromosomes, e.g., one 4CL, one HCT, and one CCR on chromosome 9, one CCoAOMT and one CAD on chromosome 12 (Fig. S16, see online supplementary material), and such gene clusters are probably beneficial for transcriptional efficiency of genes in the same pathway. Interestingly, in the same module, we can also find CesA (cellulose synthase) and IRX (Irregular Xylem 7) genes responsible for cellulose and hemicellulose biosynthesis, which also displayed an increasing pattern during pericarp development and can explain the cellulose and hemicellulose accumulation in the process. Of all genes in the module, Ssp11G009220.1 (HCT) and Ssp01G009950.1 (IRX) were estimated as hub genes with extensive connections to other genes (Table S12, see online supplementary material), suggesting that lignin and hemicellulose biosynthesis are important physiological activities during pericarp development. These results indicate the accuracy and concordance between molecular evidence and phenotypes in this study. The genes in the red co-expression module were mainly enriched in ‘RNA modification’, ‘nucleic acid phosphodiester bond hydrolysis’, ‘xylan biosynthetic process’, and ‘xylan metabolic process’ with respect to ‘biological progress’, and ‘hydrolase activity, acting on ester bonds’, ‘nuclease activity’, ‘endonuclease activity’, and ‘RNA binding’ with respect to ‘molecular function’ (Fig. S17, see online supplementary material). In addition, the XP-CLR analysis suggested that two genes associated with lignin and hemicellulose biosynthesis are under selective sweeps, i.e., Ssp03G010310.1 (F5H) and Ssp01G009950.1 (IRX7) (Fig. S18 and Table S13, see online supplementary material), suggesting the preservation of their functional roles in the pathway during evolution.

Previous studies identified a number of transcription factors (TFs) regulating lignin biosynthesis in plants [31–34], and they all belong to the R2R3-MYB family. Therefore, we took a further look into the co-expression network, and tried to identify key regulators that may govern the LBP genes during pericarp development. Firstly, phylogenetic analysis incorporating *S. xylocarpa* and *Arabidopsis thaliana* MYB genes were performed to determine all R2R3-MYBs in *S. xylocarpa* (Fig. S19, see online supplementary material), then 33 TFs closely connected to the LBP genes in the co-expression module were identified (Fig. S19, see online supplementary material), and finally they were cross-compared with the R2R3-MYBs to screen out the most promising regulators, which in our analysis, included seven genes, namely *SxMYB20* (Ssp07G007490.1), *SxMYB42* (Ssp10G023140.1), *SxMYB62* (Ssp08G018180.1), *SxMYB83* (Ssp10G002340.1), *SxMYB85* (Ssp09G003180.1), *SxMYB86–1* (Ssp09G018570.1), and *SxMYB86–2* (Ssp11G007090.1) (Fig. 5d). These TFs may be the reason that triggers the high expression of LBP genes.

## Discussion

To enhance plant conservation efforts for the threatened ornamental *Sinojackia* species, we assembled a high-quality genome of jacktree and conducted genomic resequencing of individuals in rare natural distribution habitats in Eastern China. To explore the phylogenetic positions of the jacktree and Ericales, we reconstructed a phylogenetic tree using the high-quality genome of the jacktree and another 26 angiosperms. However, our result is discordant with that of the Angiosperm Phylogeny Group IV (APG IV) [24], which recovers Cornales and Ericales as the successive sister groups to lamiids-campanulids. In fact, this discordance reflects the cyto-nuclear conflict, as plastid genome-based phylogenetic inferences mostly supported the same topology as APG IV [35–37], while recently growing nuclear evidences all favored the sister relationship between Cornales and Ericales [18, 38–40]. These data will serve as the foundation to the reconstruction of the evolutionary history and exploring the endangered mechanisms of species.

The egg-shaped fruits exhibit the distinctive morphological characteristics of *S. xylocarpa*, which are primarily appreciated for their ornamental value. The pericarp serves to protect the seeds from environmental factors and predators, which is a common preservation strategy of plants. For example, pericarp thickness is a defensive traits of *Camellia japonica* against its seed predator (*Curculio camelliae*) [41]. Similarly, it is very possible that the thickened *S. xylocarpa* pericarps might have played a protective role for seeds during the glacial age. However, it might become a factor limiting seed germination after the glacial period, as the seed germination is thus limited by the mechanical constraints of its pericarps [3]. Deleterious variations were detected in the LBP genes, yet these genes have maintained their functionality and exhibit high expression levels. While deleterious mutations typically compromise gene function, these genes exhibit evidence of strong purifying selection, indicating the influence of contradicting selective forces that preserve their functional integrity. The thickened pericarps due to the highly expressed LBP genes may be adaptive during the recent glacial age, which began about two Mya and lasted till now, with multiple subglacial and interglacial periods. Thus, the evolution pressure requires the retained functions of the LBP genes, in case of the coming subglacial period.

Nonetheless, the hard and lignified fruit pericarps (Fig. 5a) likely contribute significantly to the small population and limited natural distribution of this species. Firstly, the heavy, indurate, and inedible fruits are challenging to disperse over long distances by winds or animals, and animals are unlikely to find them palatable. Secondly, during seed germination, the highly lignified fruit pericarps are difficult to break, requiring a significant biomechanical force, likely resulting in a low seed germination rate [3]. We investigated the genetic mechanism underlying the development of its hard and lignified fruit pericarps. Our biomass analytical assay reveals that *S. xylocarpa* fruits exhibit increasing levels of lignin, as well as cellulose and semi-cellulose during fruit development and ripening. These findings support our hypothesis that the woody and fibrous content of *S. xylocarpa* pericarps is negatively correlated with their germination ratio [42], and that fruits valued for their ornamental characteristics create a virtual barrier to successive seed germination in this species.

Lignin biosynthesis is a crucial biological process in woody plants, with pathway enzyme genes present in all seed plants [43], and the resulting products often accumulating in various organs (e.g., seed coats, petioles, stems, and roots) [44, 45]. However, it appears that only *S. xylocarpa* fruit pericarps specifically accumulate high levels of lignin, likely related to the high expression of some, but not all LBP genes during fruit development (Fig. 5c). Because each LBP gene has multiple copies, largely due to tandem duplications (Fig. S19, see online supplementary material), it is possible that different copies have undergone functional divergence, leading to organ/tissue-specific catalytic activities. These highly expressed copies are likely the results of subfunctionalization and neofunctionalization, and are probably responsible for lignin biosynthesis specifically in fruit pericarps.

In addition to the functionally diverged enzyme genes themselves, their specific expression in fruit pericarps may also be influenced by regulatory elements. Previous studies have shown that TFs such as MYB, NAC (NAM/ATAF/CUC), and ARF (auxin response factor) play a regulatory role in lignin biosynthesis [46, 47]. For instance, Ding *et al.* found that MYB9, MYB60, and MYB91 might participate in the regulation of PAL, C4H, 4CL, CCoAOMT, and COMT in the seed coat of *Brassica napus* L. [48]. Through co-expression network analysis, we identified seven *R2R3-MYB* genes that are closely connected to the highly expressed LBP genes in *S. xylocarpa* fruit pericarps, suggesting they may function as potential regulators for these genes. Another explanation for the high expression of LBP genes could be attributed to the physical arrangement of upstream and downstream genes in the same signalling pathway, as they often form gene clusters, which is proposed to be functionally beneficial to transcriptional efficiency. Coincidentally, we observed the two clustered loci, collectively comprising five LBP genes (one 4CL, one HCT and one CCR on chromosome 9, one CCoAOMT and one CAD on chromosome 12, Fig. S16, see online supplementary material), and more importantly, these LBP genes are highly expressed in fruit pericarps (Fig. 5c), which strongly supports our hypothesis.

While the germination barriers of *S. xylocarpa* seeds can be explained based on the evidence from our experiments and multi-omics analyses, the ultimate question of how to efficiently conserve this endangered plant remains unanswered. Population genomic analysis not only reconstructed the evolutionary history but also provided insights into this ultimate question. Following the divergence of Nanjing and Ningbo populations, Nanjing continued to experience population size reduction, while Ningbo has maintained (or slightly recovered) its population size (Fig. 4b), resulting in Ningbo having a larger population size and a richer genetic diversity than Nanjing today. This discrepancy between the two populations may be attributed to different habitat conditions. Nanjing is an inland city, while Ningbo is located on the coast of the west Pacific and closer to the subtropics (Fig. S7, see online supplementary material), so the warm and moist air in Ningbo might soften *S. xylocarpa* fruit pericarps, thereby facilitating the germination process. Additionally, the two sharp declines in population size of *S. xylocarpa* correlate with the decline in atmospheric surface air temperature (Tsurf) and the escalation of the Chinese loess mass accumulation rate (MAR) (Fig. 4b), suggesting that its population evolutionary history was likely influenced by climatic changes and human activities. Based on these analytical results, we propose a strategy of providing additional artificial watering to the soil for *in situ* conservation. For *ex situ* conservation, priority can be given to botanical gardens in the coast areas of South and East China. Furthermore, the two populations (Nanjing and Ningbo) were genetically separated. Artificial cross-breeding is recommended as a means to increase its genetic diversity.

The interaction between plants and microbes can be considered as another conservation strategy. Certain microbes have the ability to degrade lignin in seed coat in an environmentally friendly and efficient way [49]. For instance, specific mycorrhizal fungi are required for the seed germination of threatened *Paphiopedilum* orchids [50]. *S. xylocarpa* seeds remain buried in soils for more than two years before germination. If there are specific microbes capable of degrading lignified *S. xylocarpa* fruit pericarps, we could isolate these microbes and apply them to the soils where *S. xylocarpa* fruits are buried for further propagation. This strategy aims to improve the germination rate of *S. xylocarpa* and can be adopted together with other strategies.

In summary, this work provides essential genomic data for the endangered ornamental plant jacktree, which serves as a valuable source for studying the evolutionary history and the endangerment mechanisms of this plant. Based on this data, a proper conservation and restoration plan has been proposed [4]. The molecular basis underlying high lignification in the *S. xylocarpa* pericarps suggests a direction for screening and developing new cultivars with low lignin content fruit pericarps, which may be better candidates for re-introduction into the wild fields to further enlarge the colony of *S. xylocarpa*. Owing to the similarity in biological features (e.g., lignified pericarps) among *Sinojackia* species, this study provides a starting point for exploring the causes of endangerment in other *Sinojackia* species. Additionally, this study provides a reference strategy in conservation biology, particularly for studies exploring the mechanisms of endangered ornamental woody plants.

## Materials and methods

### Genome survey and assembly

An individual of *S. xylocarpa* was cultivated on campus at Nanjing Forestry University (118.81°N, 32.08°E), Nanjing, Jiangsu Province, China. The plant material was selected for genome survey and genome assembly, and its fresh leaves were utilized for genomic DNA isolation. For the survey of the *S. xylocarpa* genome, the extracted DNA was served as establishing two paired-end (PE) libraries, each with a 300 bp insert. Sequencing of each PE library was conducted to generate ~30 Gb reads via Illumina NovaSeq 6000. For *de novo* genome assembly of *S. xylocarpa*, the extracted DNA was subjected to sequencing on a PacBio Sequel II system to yield HiFi long-read data, averaging 17.8 kb in length. For chromosome anchoring, Illumina NovaSeq 6000 was adopted to sequence a prepared Hi-C library [51], resulting in 150 bp PE reads.

The genome survey was conducted using GenomeScope (v2.0) [52] based on 60 Gb Illumina reads from two PE libraries. To construct a genome assembly, contigs were derived from *de novo* assembly of PacBio HiFi long-reads with the assistance of hifiasm (v0.12) [53]. The resulting contigs were then anchored onto 12 pseudochromosomes by using both HiC-Pro (v2.10.0) [54] and LACHESIS [55] with Hi-C datasets. Alignment of Hi-C reads against these resultant contigs was performed using BWA (v0.7.10-r789) [56].

Genome assembly assessment was processed in term of DNA read mapped rate and annotation completeness. Mapping of Illumina reads to the *S. xylocarpa* genome assembly was carried out using BWA. CEGMA (v2.5) [57] and BUSCO (v4.0.6) [58] were employed to appraise the gene completeness within the *S. xylocarpa* assembly, with results summarized in Tables S14 and S15, respectively (see online supplementary material).

### Genome annotation

The *S. xylocarpa* genome was explored for protein-encoding genes searched with a synergistic application of transcriptome-based, sequence similarity-based and *de-novo* predicting approaches. In the transcriptomic evidence-based prediction method, GeneMarkS-T (v5.1) was utilized to infer the gene models from the transcripts of reference-guided assembly. This process involved alignment of RNA-Seq reads against the *S. xylocarpa* genome by HISAT2 (v2.0.4) [59], followed by genome-based assembly with using StringTie (v1.2.3) [60]. For the homology-based technique, these reference gene models were drawn from four species, namely *Actinidia chinensis*, *A. thaliana*, *R. simsii*, and *Nyssa sinensis*, using GeMoMa (v1.7) [61]. For *ab initio* prediction methods, the *de-novo* gene models were archived by a combination of Augustus (v2.4) [62] and SNAP from Korf Lab’s GitHub. Finally, the above three sets of gene models were merged using the EVidenceModeler (v1.1.1) [63], followed by refinement with PASA (v2.0.2) [64].

Protein-encoding genes were aligned and annotated with Diamond (v0.9.24) [65], with searches conducted on NCBI NR (Non-redundant) database, as well as SwissProt/TrEMBL and EggNOG (v5.0) [66]. Additionally, HMMER (v3.1) was adopted to delineate protein domains by searching against Pfam (v33.1) [67].

Non-coding RNAs (e.g., miRNA, rRNA, tRNA, snoRNA, and snRNA) were determined in the *S. xylocarpa* genome, and the utilized methods and databases were summarized in Table S16 (see online supplementary material). Transposon elements (TEs) were identified in the *S. xylocarpa* genome using a combination of RepeatModeler and RepeatMasker under RepBase database (v19.06). Annotation of terminal repeat retrotransposons (LTR-RTs) in the *S. xylocarpa* genome was accomplished using both LTRharvest [68] and LTR_finder [69]. TRF (v4.09.1) from Benson Lab’s GitHub and MISA (v2.1) were utilized to delineate tandem repeats in the *S. xylocarpa* assembly.

### Phylogenetic analysis and estimation of divergence time

OrthoFinder (v2.5.4) [70] was applied to delineate orthologous gene clusters between *S. xylocarpa* and 26 other angiosperms. The low-copy orthologous genes were screened based on three criteria: (i) presence in ≥75% of genomes, (ii) ≤5 copies per orthologous gene group per species, and (iii) selection of the longest copy for phylogenetic analysis. A total of 1291 low-copy orthologous genes were predicted between them, and 13 145 homologous groups were identified in *S. xylocarpa*. Multiple sequence alignment (MSA) was accomplished using low-copy orthologous proteins via MUSCLE (v3.8.1551) [71], followed by constructing a well-supported maximum likelihood (ML) tree by IQ-TREE (v1.6.12) under the best-fitting model (JTT + F + R6) [72]. The MCMCTree program belonging to the PAML (v4.9) [73] package was utilized to date the divergence times of *S. xylocarpa* and the remaining 26 angiosperms. Three dated ages were chosen from TimeTree3 as standard normal priors, aligning with the speciation intervals for *A. thaliana* and *Vitis vinifera* (109–123 Mya), *S. xylocarpa* and *Souroubea exauriculata* (89–118 Mya), and *Ardisia humilis* with *Primula veris* (42–79 Mya). CAFE 5 [74] was employed to deduce the expansion and contraction patterns occurring in orthologous gene families across *S. xylocarpa* and the 26 other angiosperms.

### Whole-genome duplication (WGD) events investigatory and ancestral karyotypes reconstruction

WGD software [75] was utilized to delineate the distribution of paralog ages, as indicated by synonymous substitutions per synonymous site (*Ks*) values. The MCL package was adopted to reconstruct gene family memberships using all potential paralogs which were deduced via all-vs-all protein BLAST [76], imposing an e-value of 10^−10^. MAFFT [77] was exploited for multi-alignment of each family. FastTree [78] was chosen to delineate a phylogenetic tree per gene families with n*(n-1)/2 ≤ ‘max airwise’. CODEML implemented in the PAML (v4.9) package [73] was employed to calculate ML-based *K*s values for each gene pair. Mixture modelling was done for all inferred WGDs with the BGMM (Bayesian Gaussian Mixture Models) method. The WGDI (v0.6.2) [79] was employed to achieve collinear segment pairs. All syntenic blocks were determined with WGDI under ‘*P*-value = 0.05’ and the improved collinearity mode. The *K*s pipeline in WGDI was adopted to infer the *K*s value for each anchoring gene pair within a syntenic block, and the block mode was utilized to draw the *K*s dotplot of all anchor pairs. The KsPeaks pipeline in WGDI was applied to delineate the *K*s median value for each syntenic block. Finally, the *K*s distributions were summarized and visualized using the R package ggplot2 (v3.5.0).

The pattern of chromosomal evolution within the order Ericales was reconstructed by utilizing well-defined polyploidization events and established phylogenetic relationships. Initially, we utilized WGDI [79] based on adjacent conserved collinear blocks to facilitate intra- or inter-genome comparisons, resulting in collinear dotplots annotated with Ks values. By including suitable outgroups and applying maximum parsimony rooted in well-defined phylogenetic relationships, we reconstructed ancestral karyotypes at every node within the phylogenetic tree of the Ericales. Lastly, by juxtaposing the acquired ancestral chromosomes with present-day species and elucidating the chromosomal evolution pattern of the Ericales.

### Scanning electron microscopy and determination of lignin content

The drupes of *S. xylocarpa* at six developmental stages were harvested from trees located at the Xinzhuang campus of Nanjing Forestry University (118.81° N, 32.08° E). The drupes were collected every 1 month from April to September 2022, and the harvested samples were quickly frozen in liquid nitrogen and subsequently transferred to a −80°C refrigerator for storage. The content of lignin at six developmental stages was measured using a JC2203-M kit (JC DTECT Biotechnologies Co., Ltd, Nanjing, China) with triplicate samples per stage.

The fruits in the ripening stage were harvested for their morphological observation. The drupe samples were sliced into 20 μm thick sections consisting of exocarp, mesocarp, and endocarp, using a TU-213 sliding microtome (YAMATO, Saitama, Japan). A 1% aqueous Safranine O solution was utilized to stain the 20 μm-thick slices. The photographs of these qualified samples were acquisited on a BX51 microscope (Olympus, Tokyo, Japan), and processed with STL-IMCS software.

### Transcriptome sequencing of pericarps and evolution analysis

The drupes were collected at six developmental stages with three biological replicates from *S. xylocarpa* trees cultivated at Nanjing Forestry University’s Xinzhuang campus (118.81°N, 32.08°E). Illumina RNA-Seq libraries were prepared from the RNAs that were isolated with RNAprep Pure Plant Kit (Tiangen, Beijing, China) and their sequencing was accomplished on Illumina NovaSeq 6000.

Raw reads were trimmed to remove adaptors, and low-quality reads (the reads with N ratio greater than 10% or whose base with Phread quality score [Q] ≤10 accounts for more than 50% of the whole reads) were discarded. These trimmed reads were mapped against the *S. xylocarpa* genome using Bowtie2 [80]. The calculation and normalization of gene expression levels were conducted via the FPKM (Fragments Per Kilobase of exon model per Million mapped fragments) method [81]. RSEM software [82] was then used to perform the calculation of FPKM values. Gene expression heatmaps were visualized using TBtools [83]. BLASTP [76] was used to identify lignin synthase genes (including *PAL*, *C4H*, *C3H*, *F5H*, *4CL*, *CCR*, *CAD*, *COMT*, and *CCoAOMT*) from the protein sequences of *S. xylocarpa*. The lignin synthase protein sequences of *A. thaliana*, used as the query, were obtained from Uniprot [84, 85] (Table S17, see online supplementary material). The protein sequences of lignin synthases from *A. thaliana* were aligned against all the protein sequence of *S. xylocarpa*, with an e-value <10^−5^, to obtain the potential lignin synthase protein sequences of *S. xylocarpa*. The conserved domains and conserved motifs in these candidate lignin synthase of *S. xylocarpa* were identified and inspected using TBtools and CD-search. To identify *MYB* candidates in *S. xylocarpa*, we downloaded the Hidden Markov Model (HMM) profile of MYB (PF00249) and used it as the query (*P* < 0.001) to search the *S. xylocarpa* protein sequences. A BLASTP search using *A. thaliana* MYB sequences from TAIR (https://www.arabidopsis.org/) as queries was accomplished with an e-value <10^−5^. The *MYB* family was finally determined by the protein sequences of the conserved domain. ClustalW was chosen for MSA [86]. IQ-TREE2 was hired to build ML trees under the ‘-alrt 1000 -B 1000’ parameter [87].

### Genome resequencing and SNP calling

To conduct the population genomic analysis within the *S. xylocarpa* population, leaves of 20 wild samples were collected from Southeast China for genome resequencing. The natural distribution information for these samples is provided in Table S18 (see online supplementary material). Genome resequencing was conducted on the DNBSEQ-T7 sequencer to generate 150 bp PE reads with an average depth of ~18.6×.

All raw reads were trimmed with the software fastp (v0.12.4) [88] to eliminate low quality bases and adaptors, and the clean reads were then mapped to the *S. xylocarpa* genome using bwa-mem (v0.7.17) [89]. The SAM format files were processed using SAMtools (v1.15.1) [90] for sorting and merging. Picard (v2.25.0) was used for assigning read group information, including library, lane, and sample identity. GATK (v4.2.0.0) [91, 92] was adopted to predict SNPs. Subsequently, all these SNPs were annotated by ANNOVAR [93] with the ‘—neargene 2000’ option to define the length of upstream and downstream regions’ surrounding genes. The subsequent analyses were conducted using a set of 43 817 152 high-quality SNPs that had been identified. We utilized Plink (v1.90b6.21) for performing PCA (principal component analysis) and evaluating genomic diversity (π) [94]. We inferred the construction of the neighbour-joining (NJ) phylogenetic tree using Phylip (v3.697) [95] based on SNPs from genomic resequencing of 20 individuals. Population ancestry information was then calculated by Admixture (v1.3.0) [96], setting the K values from 1–6 for different numbers of clusters. PopLDdecay was employed to delineate the pattern of LD decay [97]. Long runs of homozygosity (ROHs) were sought using Plink (v1.90b6.21) within 50-SNP windows, where no heterozygous markers were allowed. ROHs were detected in regions longer than 10 kb with a minimum of 50 SNPs. ROHs were classified into different categories based on their lengths [98].

### Demographic inference and identification of deleterious mutations

The PSMC model was employed with the settings [-N25 -t15 -r5 -p ‘4 + 25*2 + 4 + 6’] to depict ancient demographic history [99]. The mutation rate (u) was estimated using the formula u = Ks/2 T. The estimated divergence time (T) was about 62 Mya between *S. xylocarpa* and *G. urceolata*. The generation time was assumed to be 10 years based on the average time taken from seed to seed, as observed, and thus u = [0.31/ [(2 × 62e^6^)] × 10] = 2.5 e^−8^ (the substitutions per site per generation). To assess the accumulation of extreme deleterious mutations in the scale tree, the ‘-lof’ parameter of SNPEFF was used to annotate SNPs that lead to loss of function (LOF). XP-CLR (v1.0) was used to estimate the XP-CLR score for detecting signals of selective sweeps in the *S. xylocarpa* genome between two populations, Nanjing and Ningbo [100].

The joint site frequency spectrum (SFS) of *S. xylocarpa* from NB and NJ was utilized for estimating the parameters of evolutionary scenario. Various models of historical events were applied to the joint SFS of NB and NJ (Fig. S12, see online supplementary material). For convergence assurance, each model underwent 50 runs with varying starting points and the model with the highest likelihood was selected. The fastsimcoal2 program was adopted to analyse the gene flow between two subpopulations under the parameters [-m -0 -C 10 -n 100 000 -L 40 -s0 -M -q] [101]. Additionally, environmental adaptation analysis between two genetic groups (NB and NJ) was conducted using latent factor mixed models (LFMM) [102] and redundancy analysis (RDA) [103]. Nineteen climatic factors were downloaded from WorldClim (https://worldclim.org/) and 27 soil-related environmental variables were acquired from the National Earth System Science Data Center (https://www.geodata.cn).

## Supplementary Material

Web_Material_uhae166
