# Supplementary figures and images for: The jacktree genome and population genomics provides insights for the mechanisms of the germination obstacle and the conservation of endangered ornamental plants
Source: Hortic Res. 2024 Jun 18;11(8):uhae166. doi: 10.1093/hr/uhae166 (PMC11300842; doi:10.1093/hr/uhae166)

# GenomeScope Profile

len:1,017,771,097bp uniq:43.8%

aa:99.1% ab:0.911%

kcov:25.6 err:0.143% dup:1.4 k:19 p:2

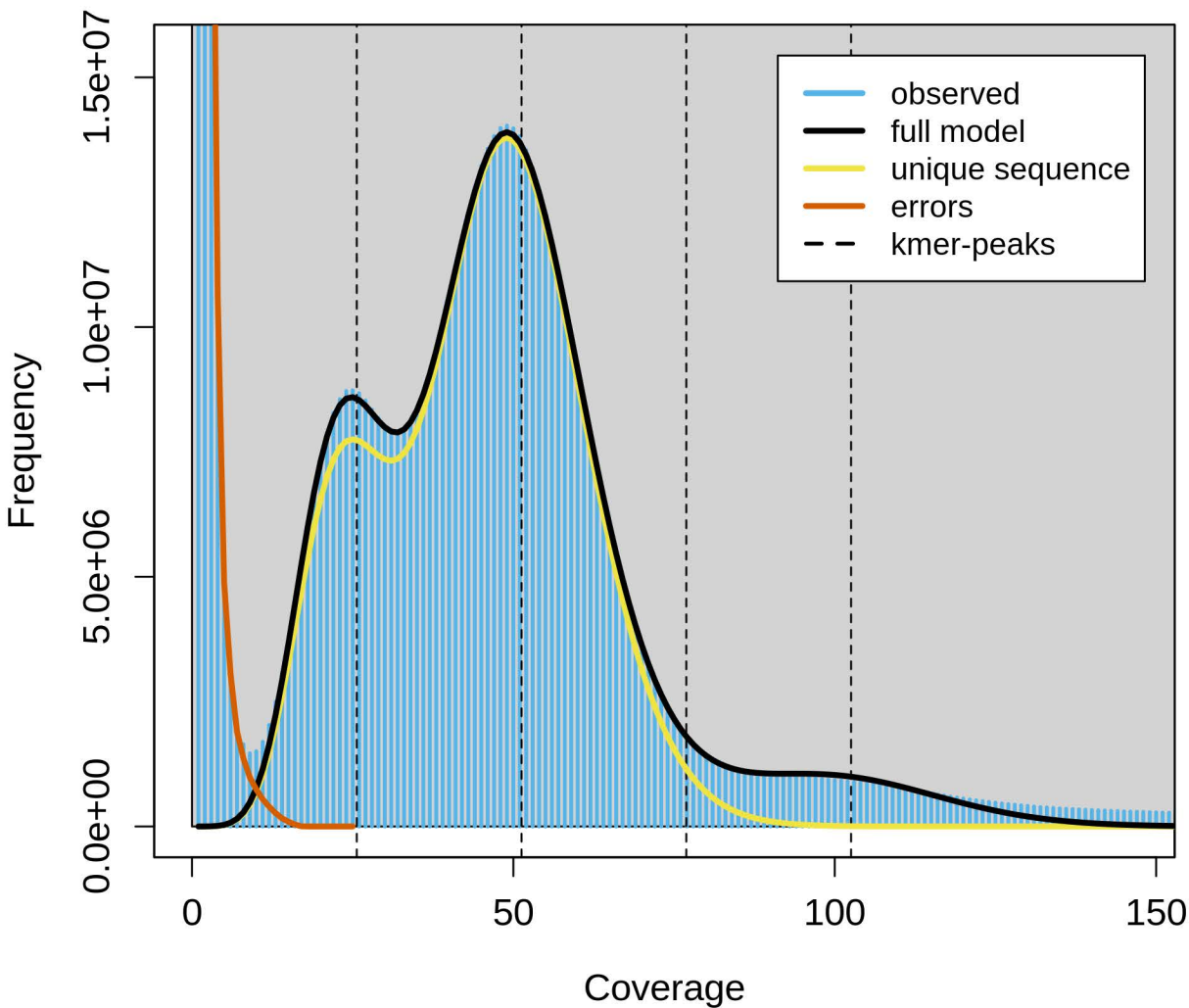

Supplement: Web_Material_uhae166 [file web_material_uhae166.zip › Figure S1. The GenomeScope plot for S. xylocarpa.pdf]

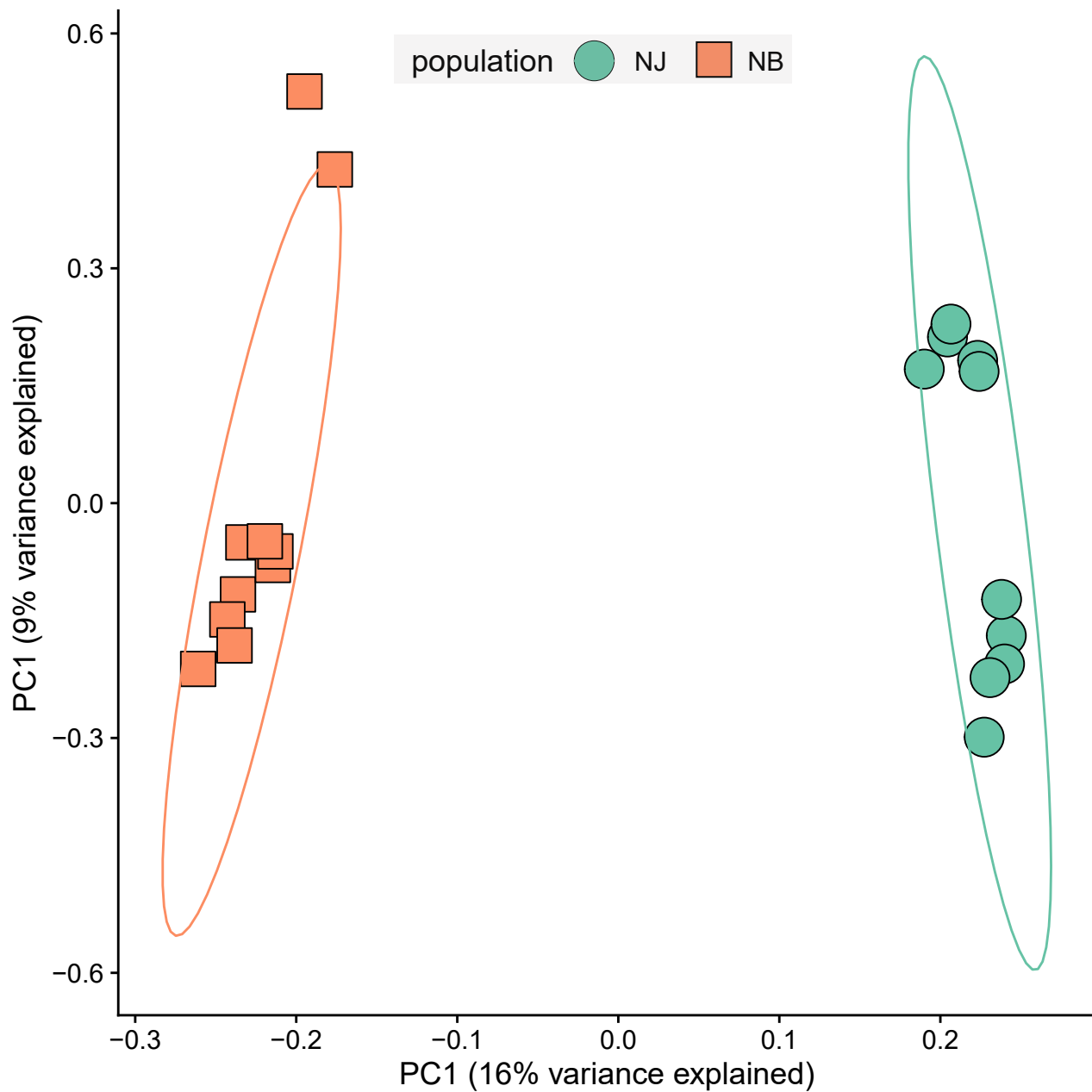

Supplement: Web_Material_uhae166 [file web_material_uhae166.zip › Figure S10. PCA analysis of genomic SNP data from the 20 sequenced samples.pdf]

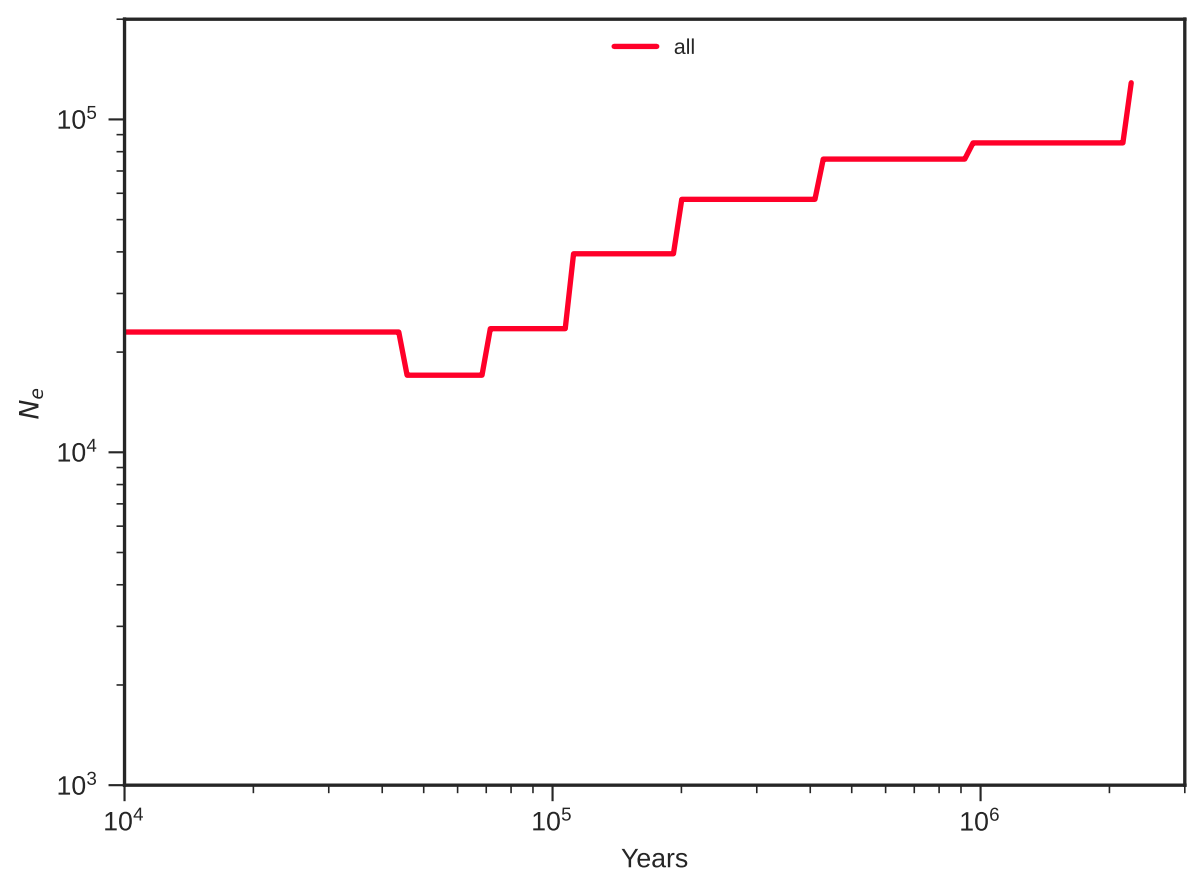

Supplement: Web_Material_uhae166 [file web_material_uhae166.zip › Figure S11. The population size histories inferred by smc++.pdf]

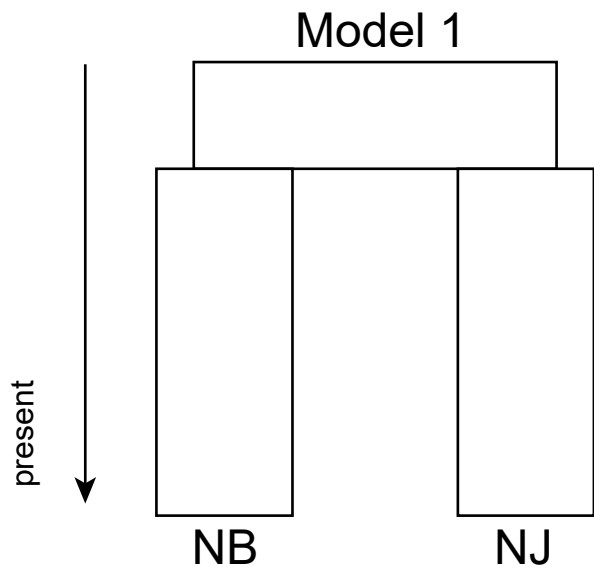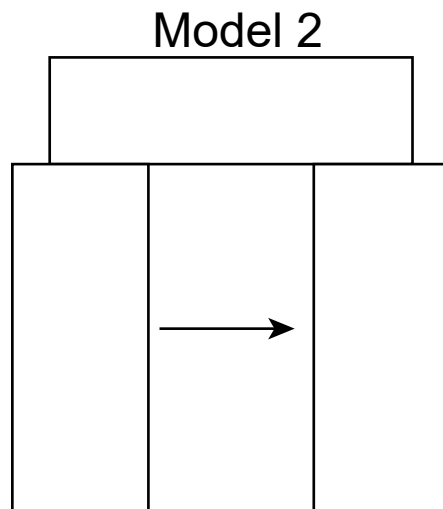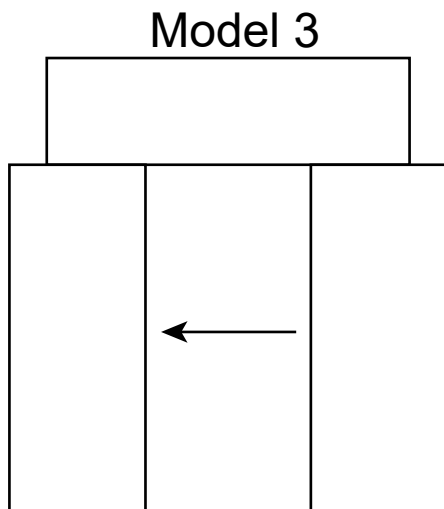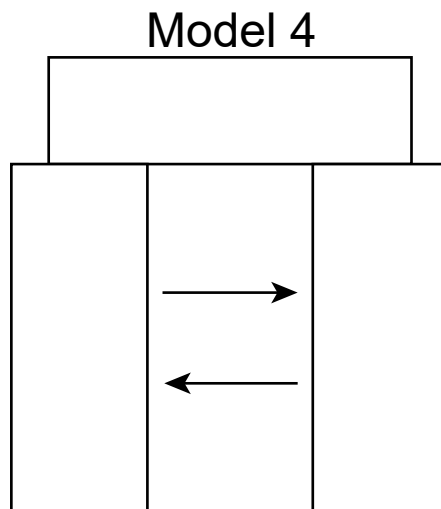

Supplement: Web_Material_uhae166 [file web_material_uhae166.zip › Figure S12. Schematic diagram of different models of S. xylocarpa demographic history in Fastsimcoal2.pdf]

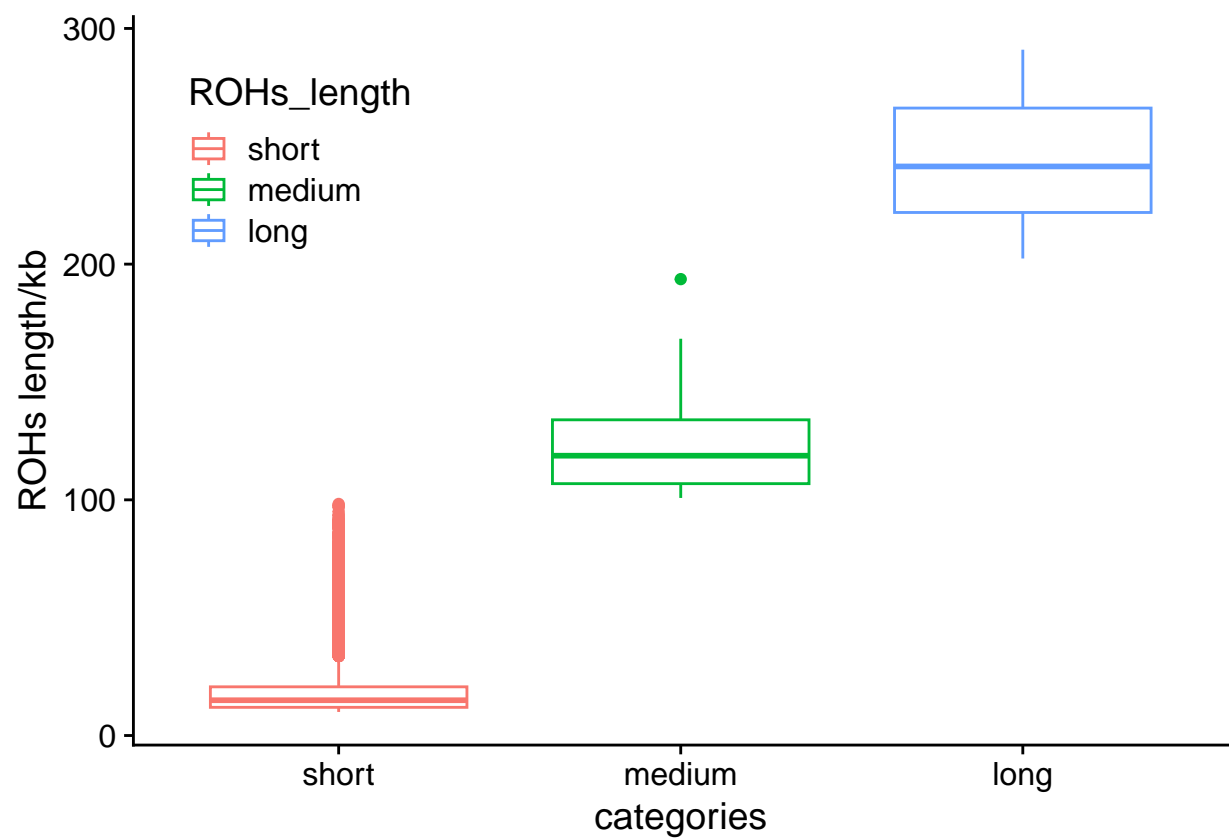

Supplement: Web_Material_uhae166 [file web_material_uhae166.zip › Figure S13. The regional distribution of ROHs.pdf]

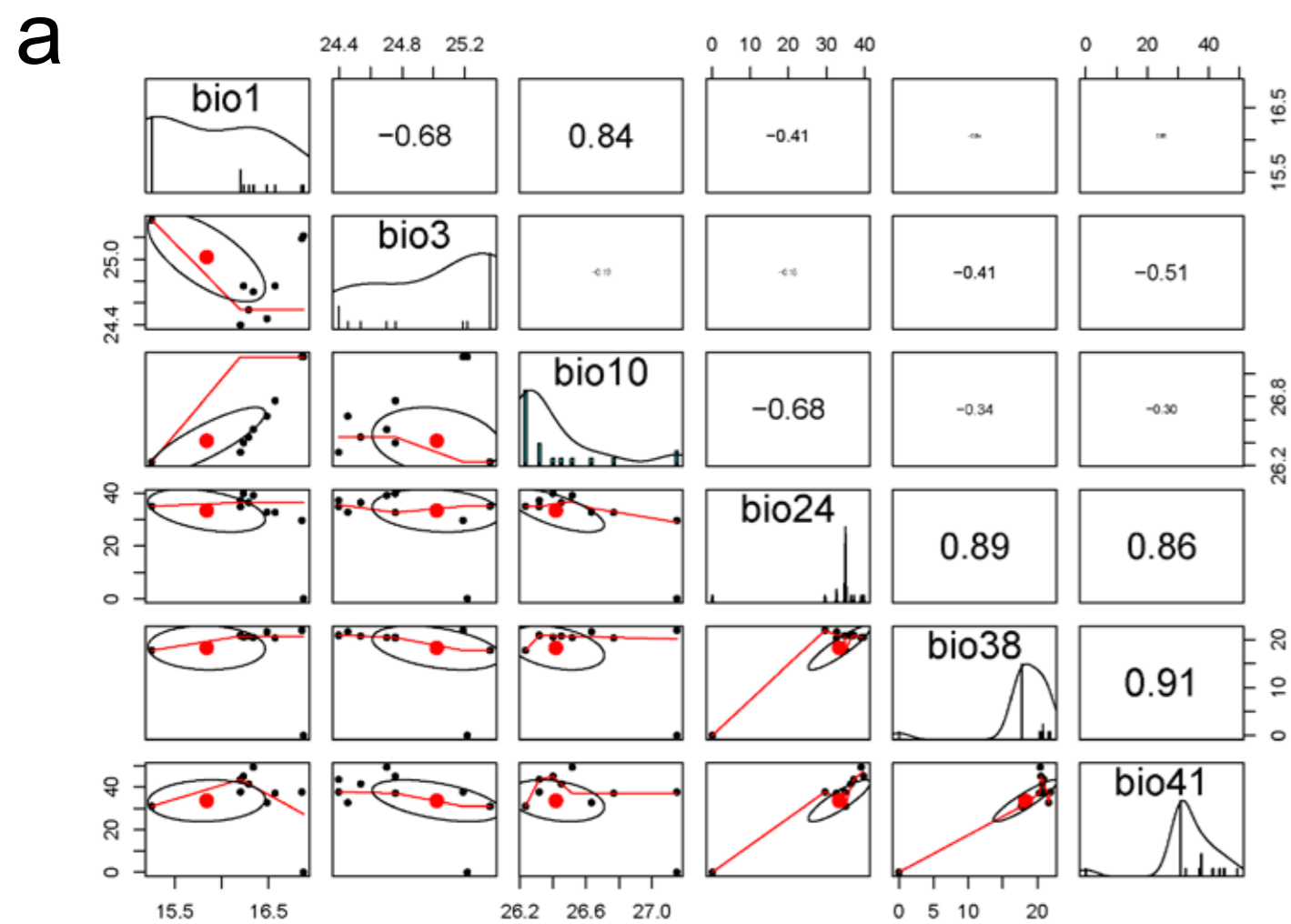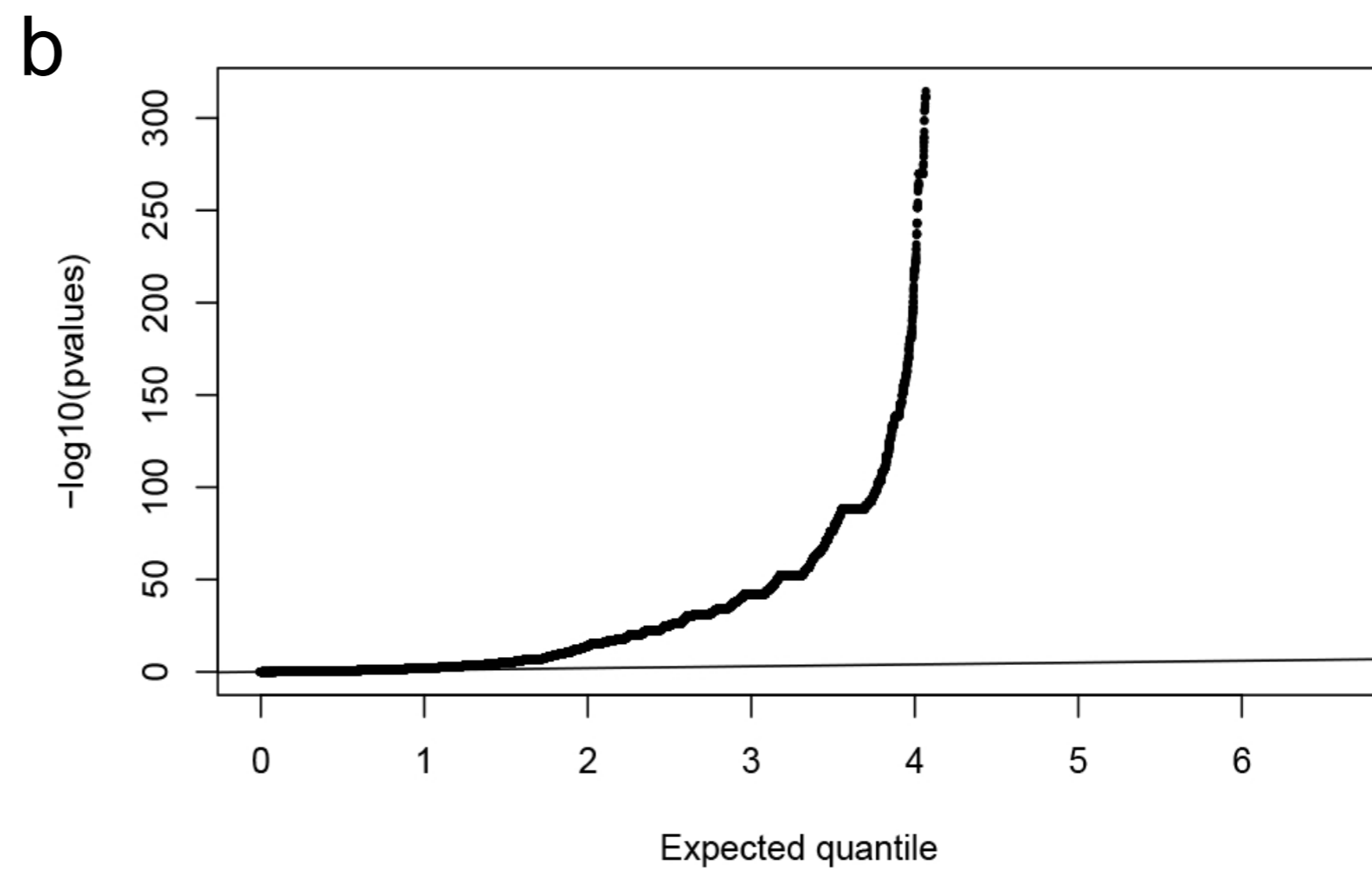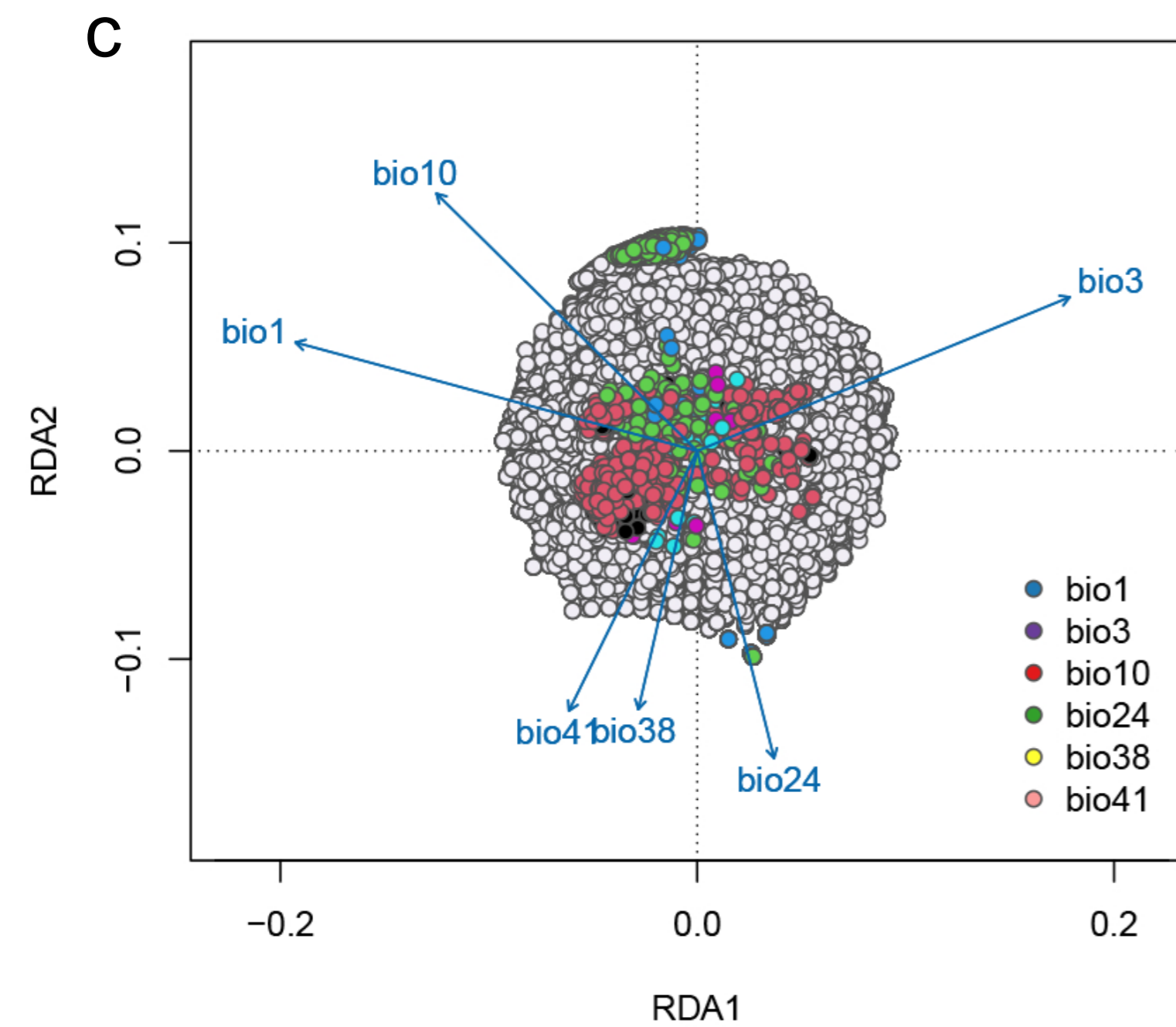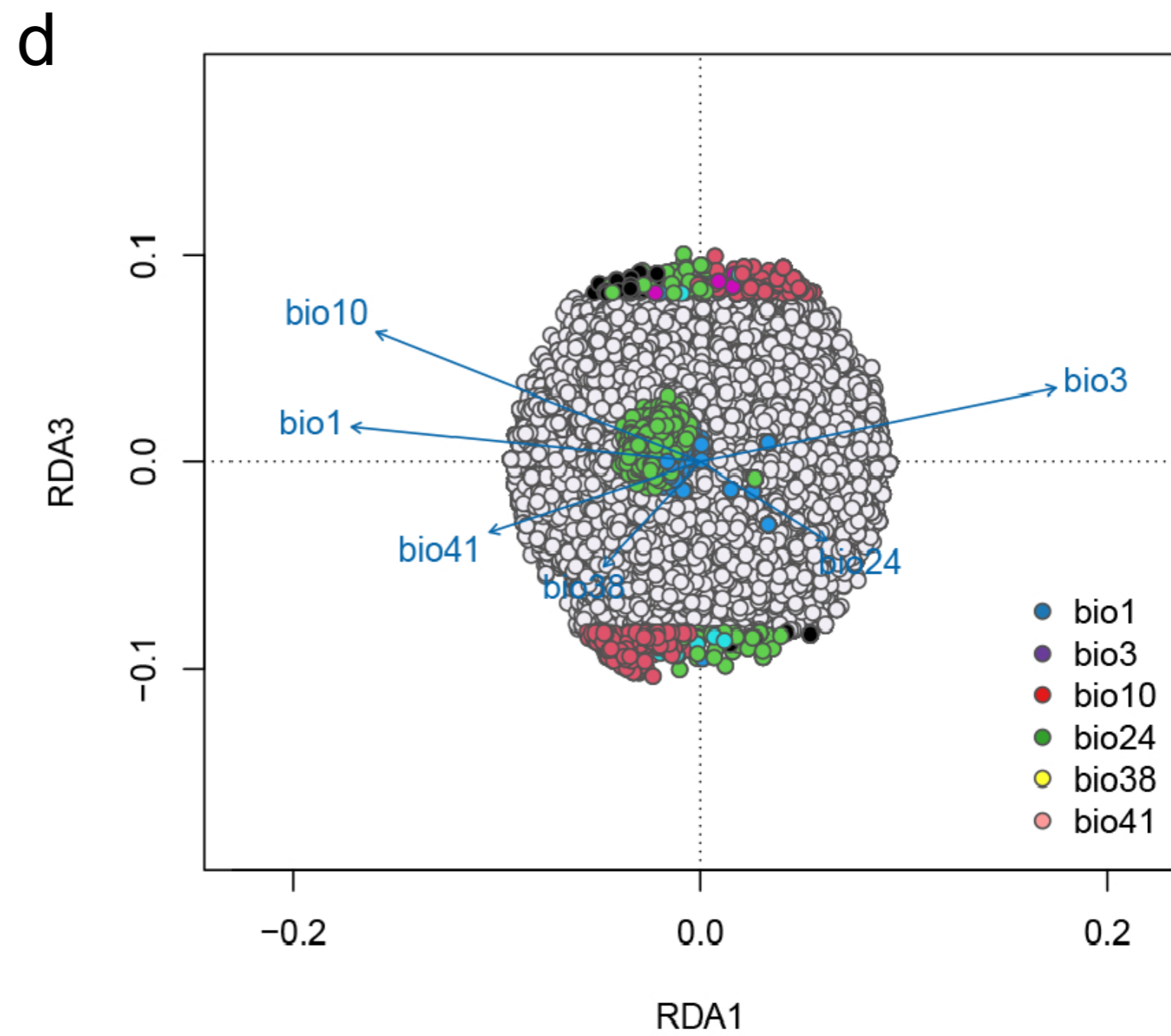

Supplement: Web_Material_uhae166 [file web_material_uhae166.zip › Figure S14 Genome-wide screening of the loci associated with local environmental adaptation.pdf]

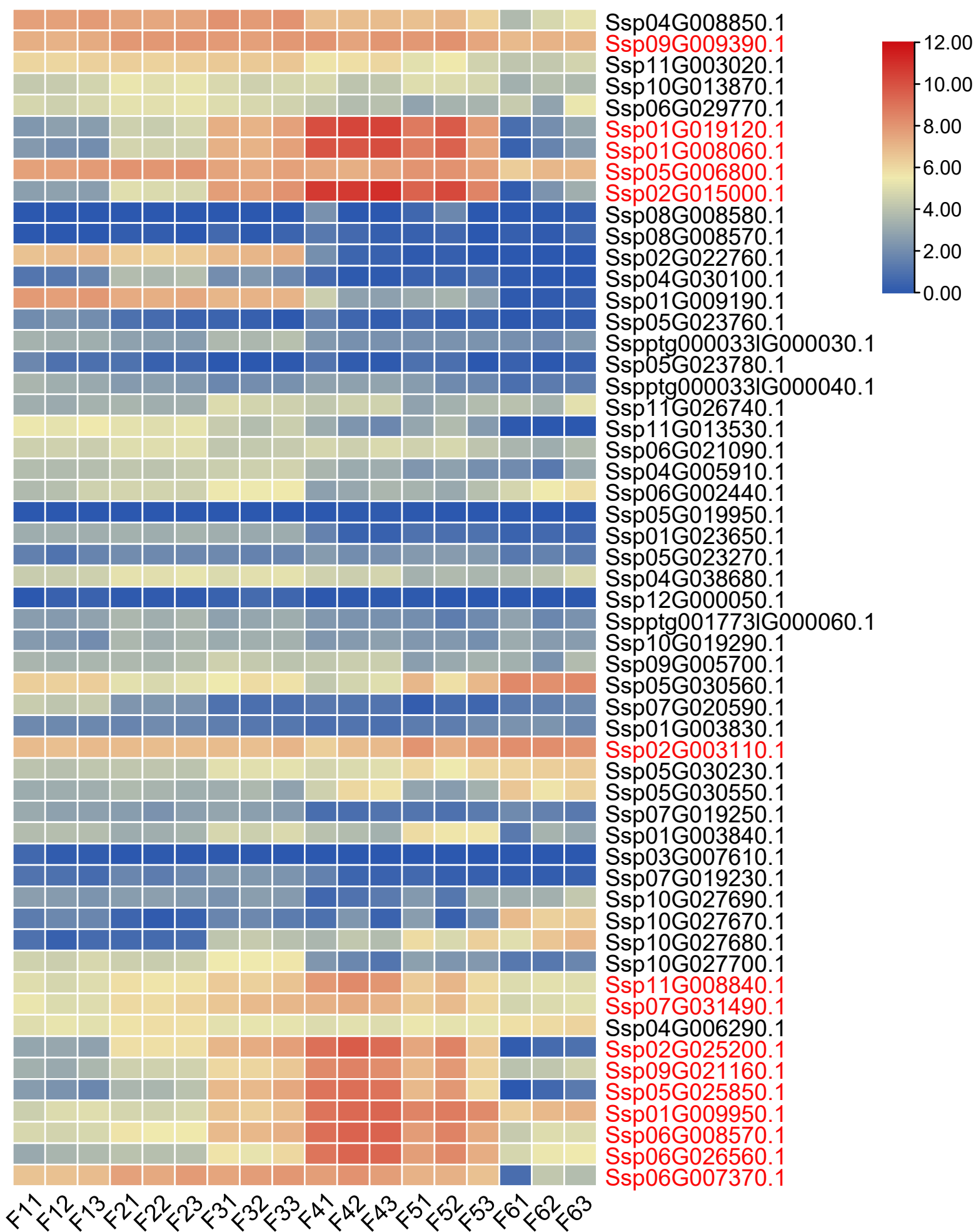

Supplement: Web_Material_uhae166 [file web_material_uhae166.zip › Figure S15. Heatmap of the gene associate with cellulose and hemicellulose biosynthesis in S. xylocarpa.pdf]

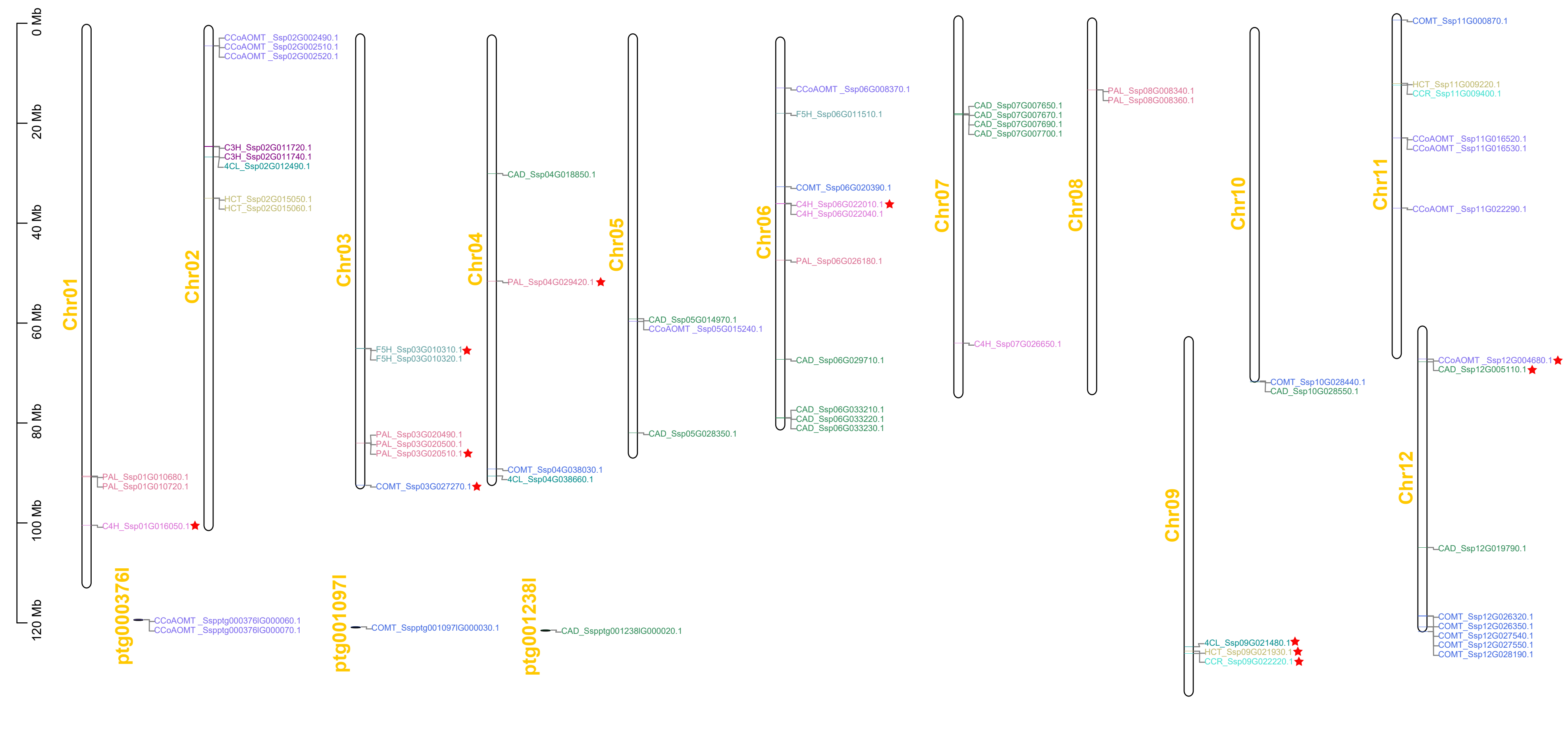

Supplement: Web_Material_uhae166 [file web_material_uhae166.zip › Figure S16. The distribution of lignin-related genes on chromosomes of S. xylocarpa.pdf]

# GO enrichment

GO term

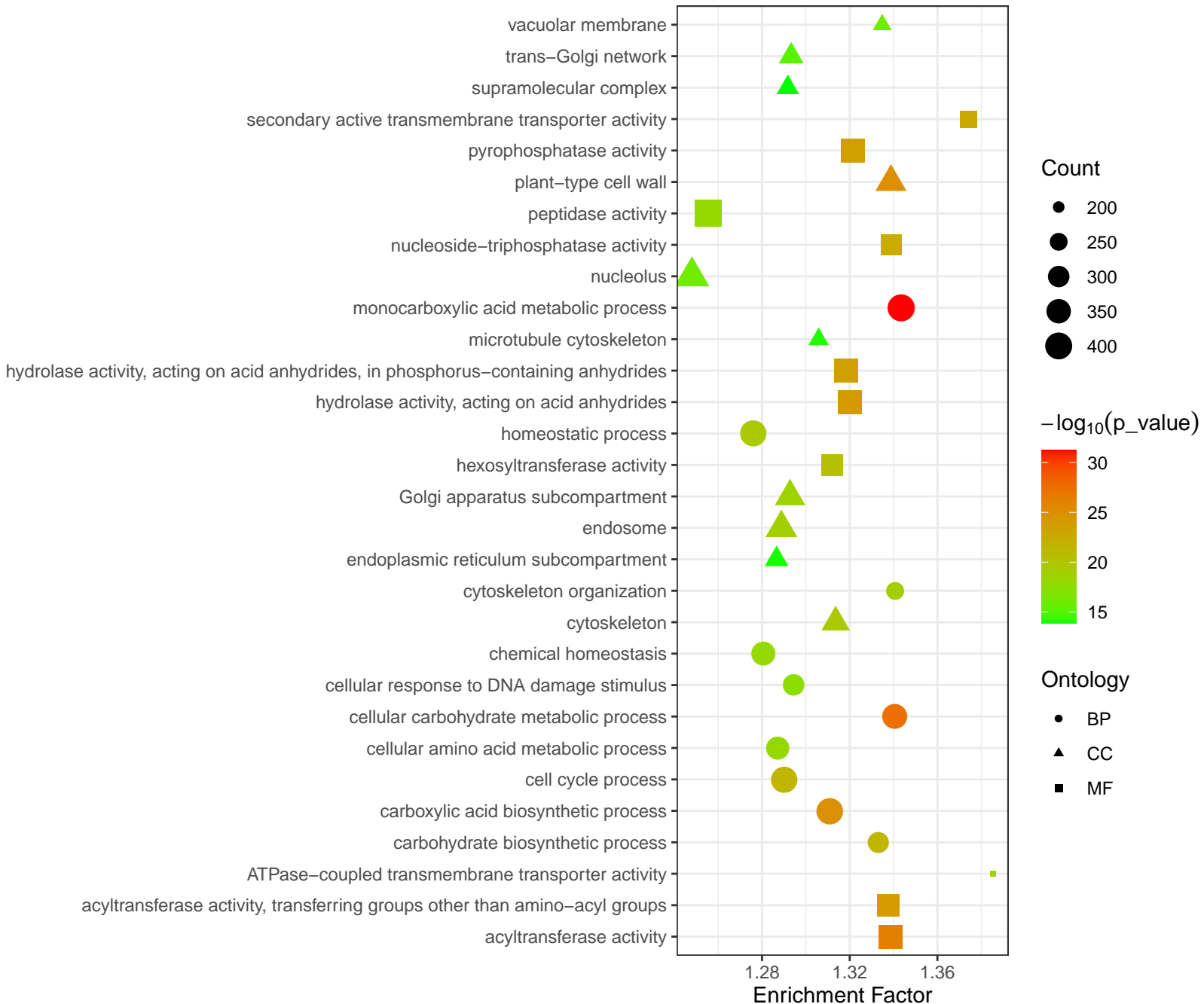

Supplement: Web_Material_uhae166 [file web_material_uhae166.zip › Figure S17. GO enrichment analysis of deleterious mutations.pdf]

a

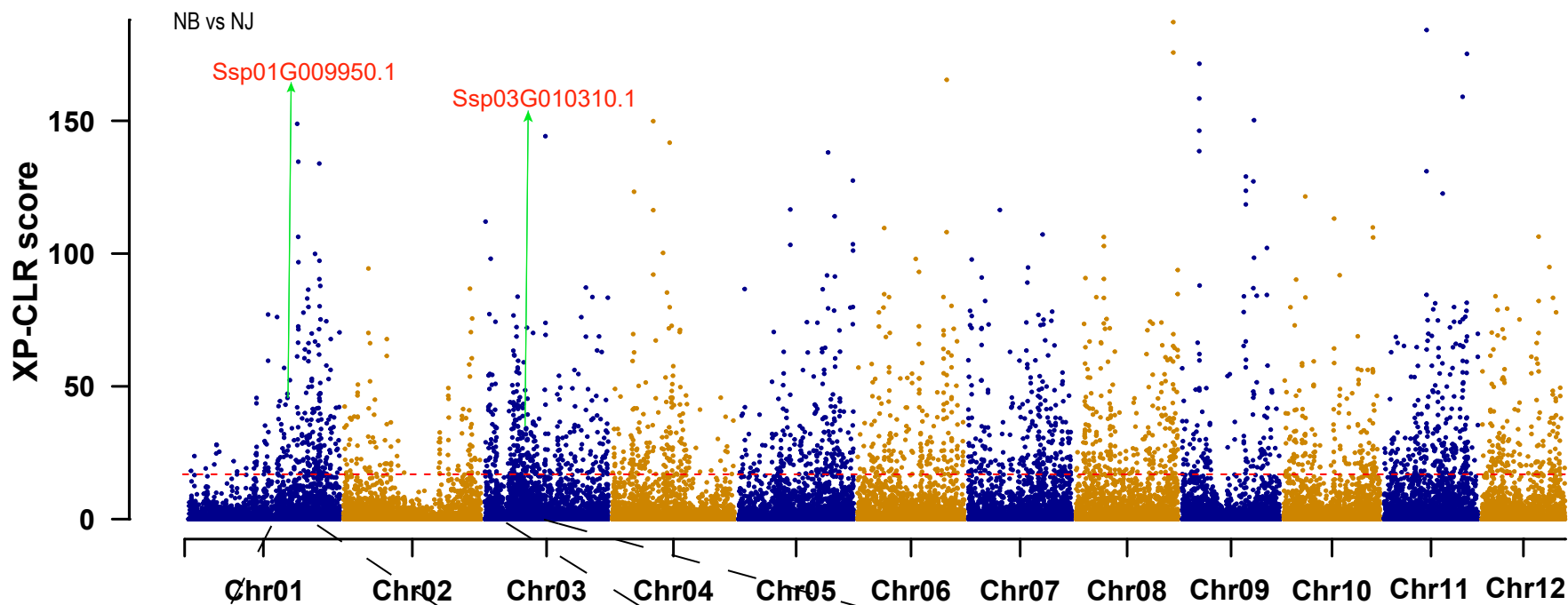

b

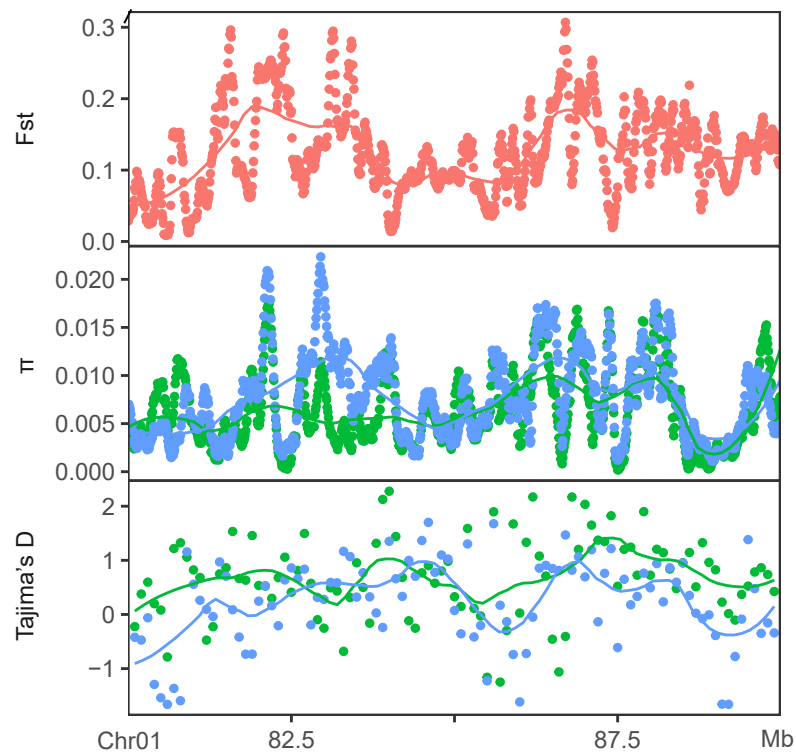

c

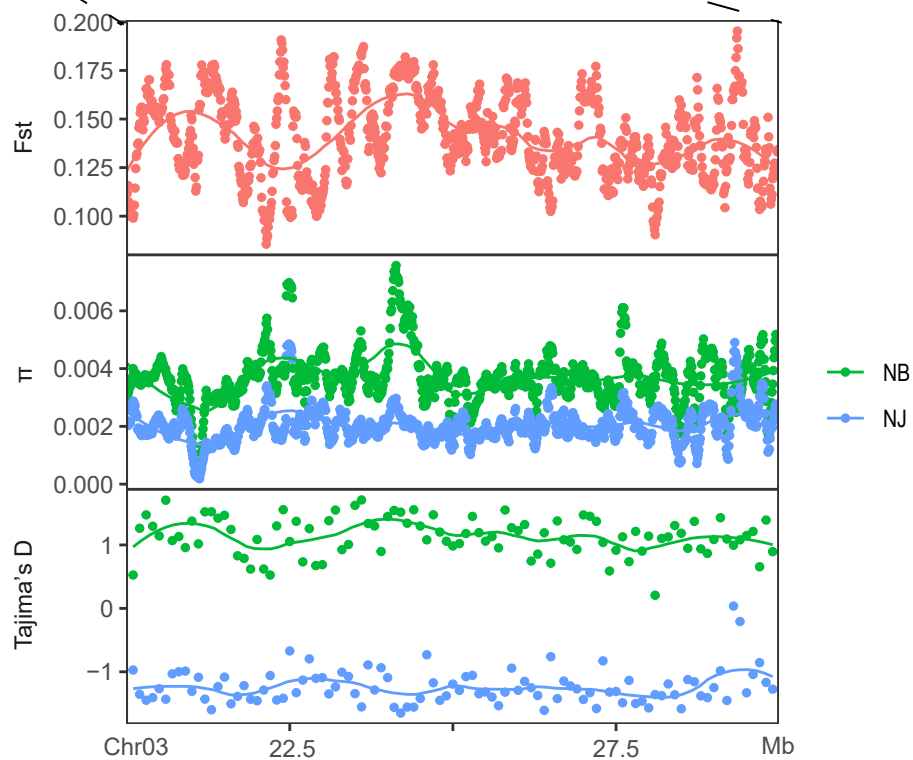

Supplement: Web_Material_uhae166 [file web_material_uhae166.zip › Figure S18. Signatures of selected signals in the genome of S. xylocarpa.pdf]

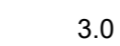

Supplement: Web_Material_uhae166 [file web_material_uhae166.zip › Figure S19. The phylogenetic tree of MYB gene family in S. xylocarpa genome.pdf]

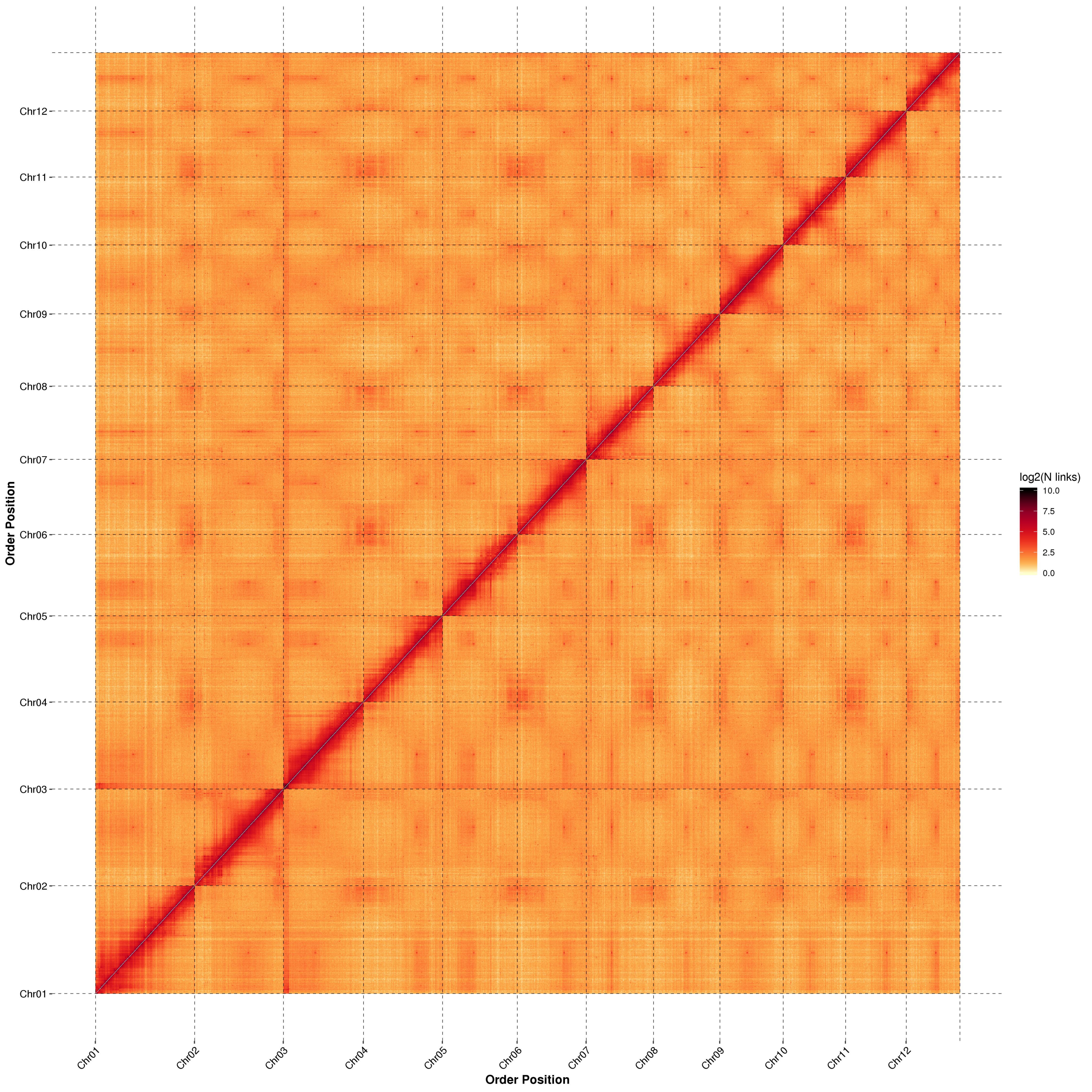

Supplement: Web_Material_uhae166 [file web_material_uhae166.zip › Figure S2. The Hi-C assisted assembly of S. xylocarpa genome.pdf]

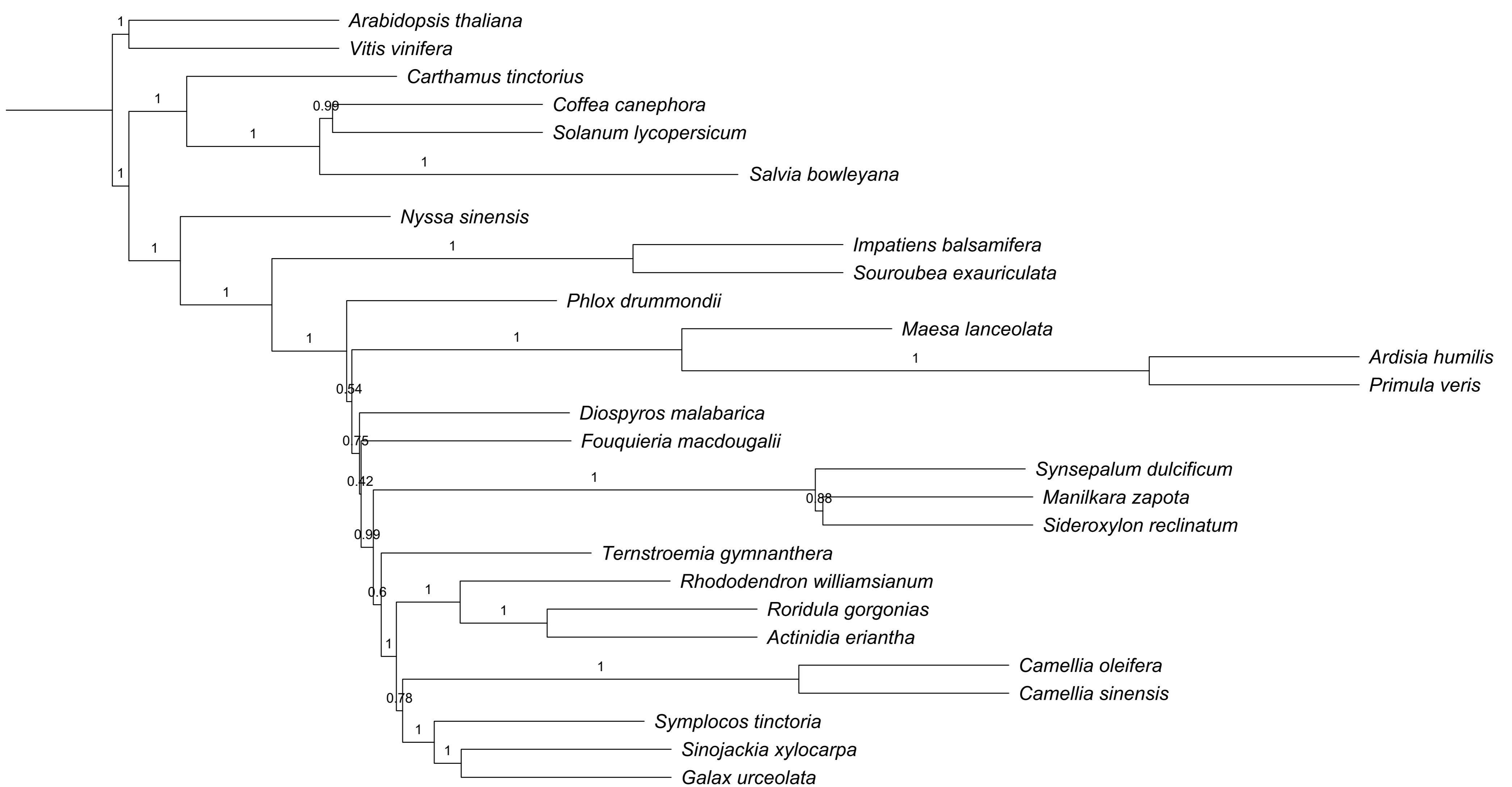

0.6

Supplement: Web_Material_uhae166 [file web_material_uhae166.zip › Figure S3. Coalescent-Based Species Tree..pdf]

■ Main topology  
■ First alternative  
■ Second alternative

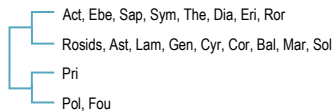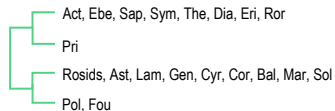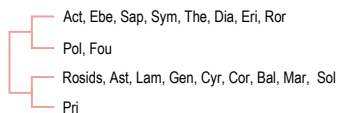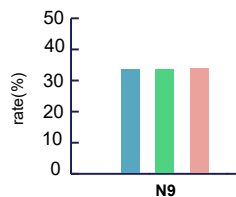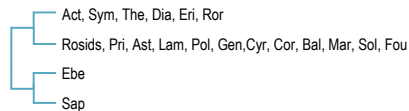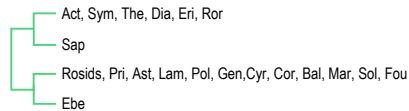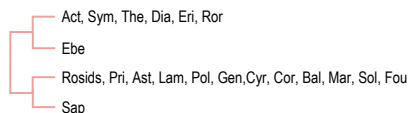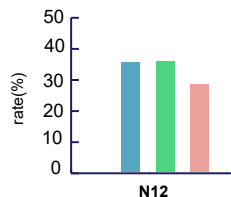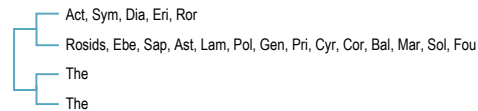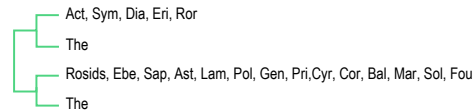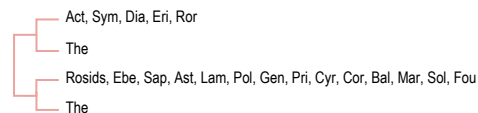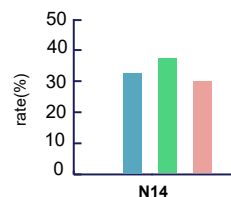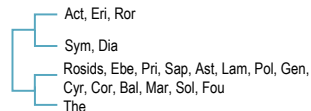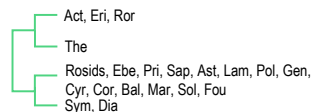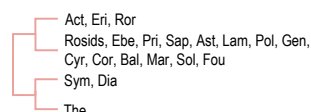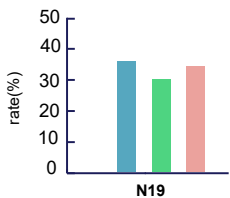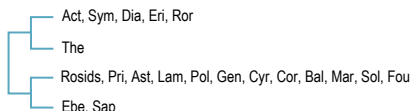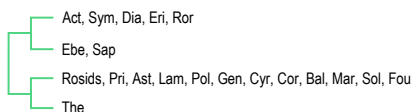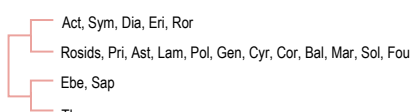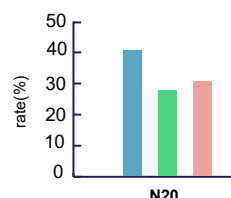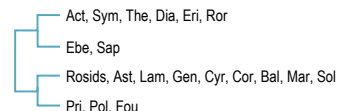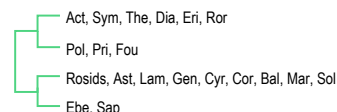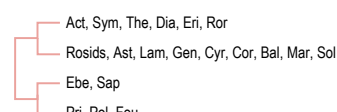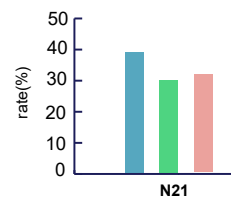

Supplement: Web_Material_uhae166 [file web_material_uhae166.zip › Figure S4. Summarized alternative topologies recovered by single gene trees.pdf]

# *Sinojackia xylocarpa*

10 9 11 4 3 7 5 12 8 2 6 1

*Sinojackia xylocarpa*

10  
9  
11  
4  
3  
7  
5  
12  
8  
2  
6  
1

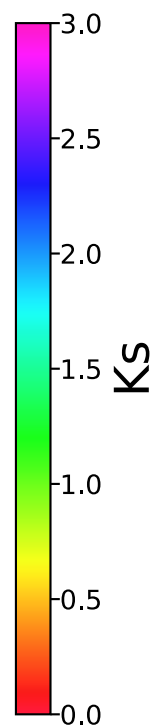

Supplement: Web_Material_uhae166 [file web_material_uhae166.zip › Figure S5. Intraspecific synteny of the S. xylocarpa genome.pdf]

# *Sinojackia xylocarpa*

*Actinidia eriantha*

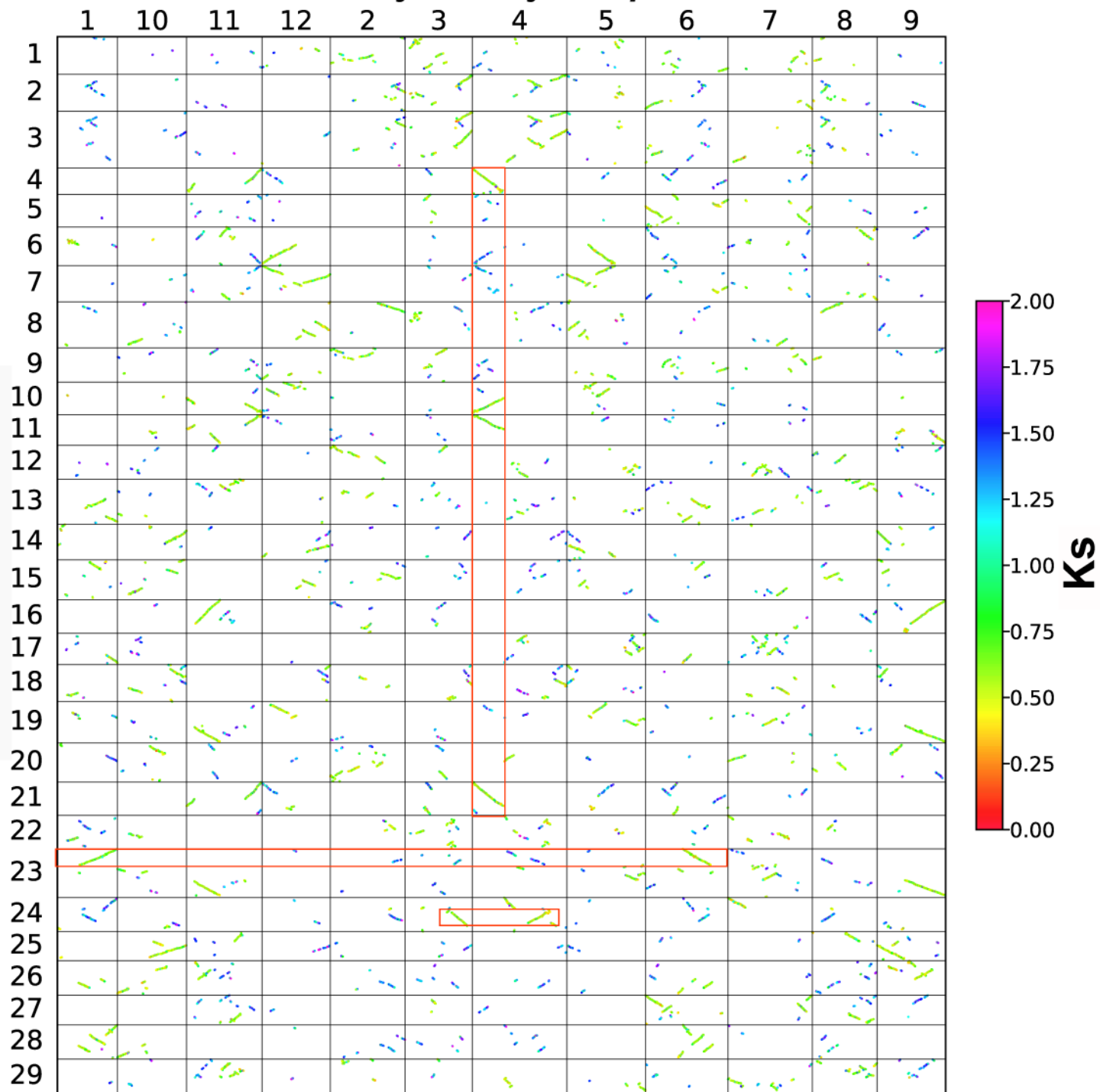

Supplement: Web_Material_uhae166 [file web_material_uhae166.zip › Figure S6. Interspecific collinearity between the S. xylocarpa and Actinidia eriantha genome.pdf]

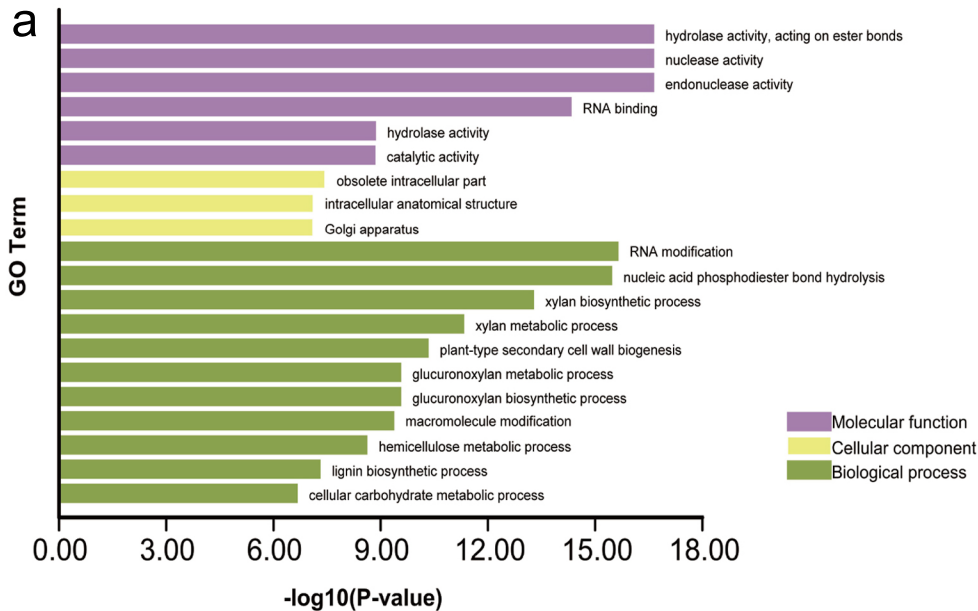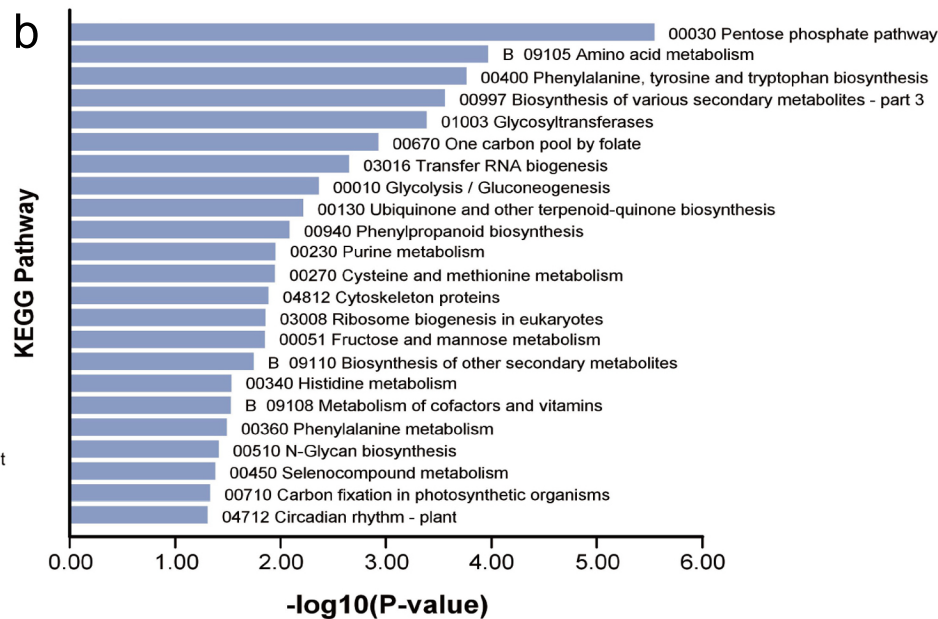

Supplement: Web_Material_uhae166 [file web_material_uhae166.zip › Figure S7. GO enrichment analysis of retained paralogous genes.pdf]

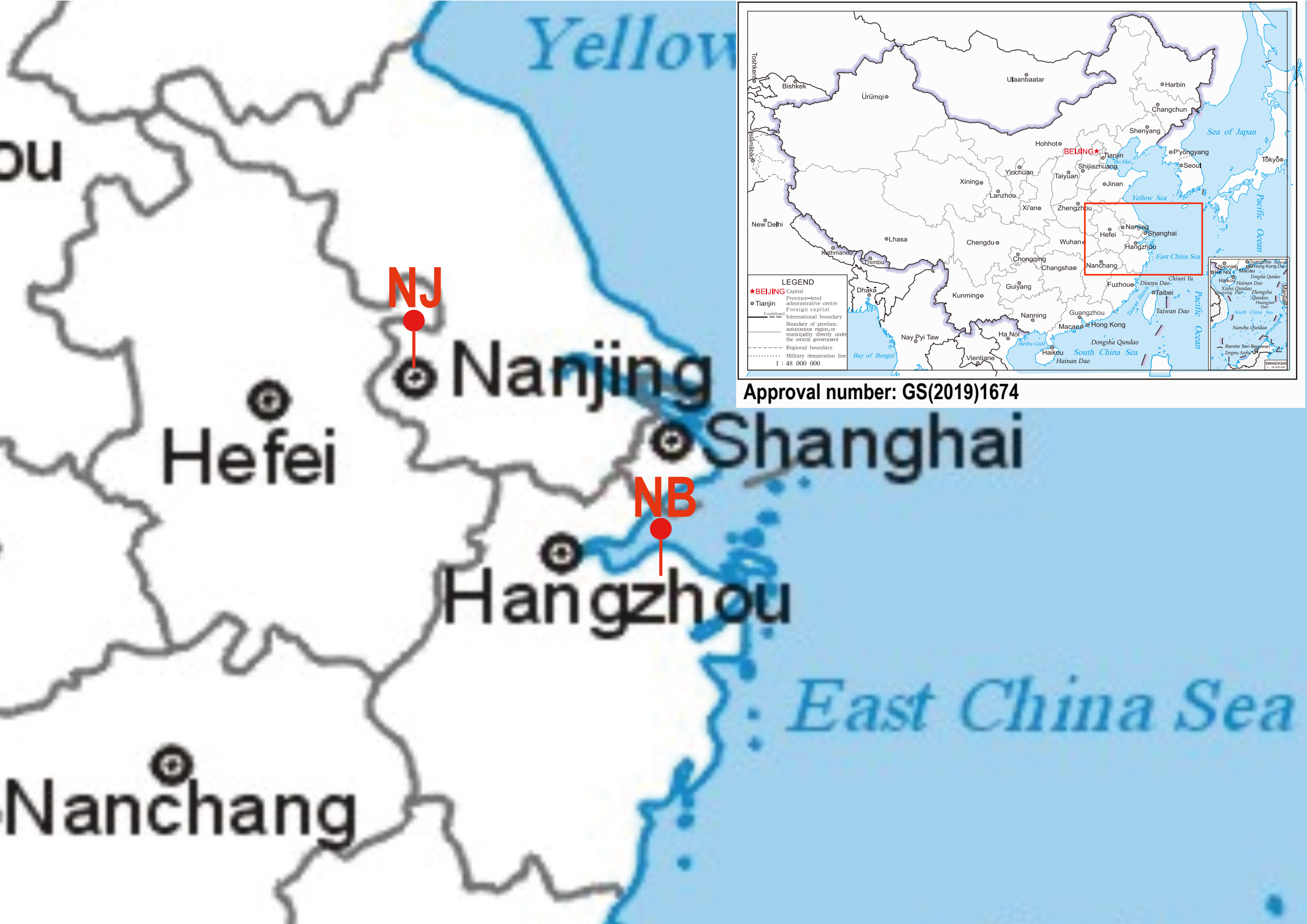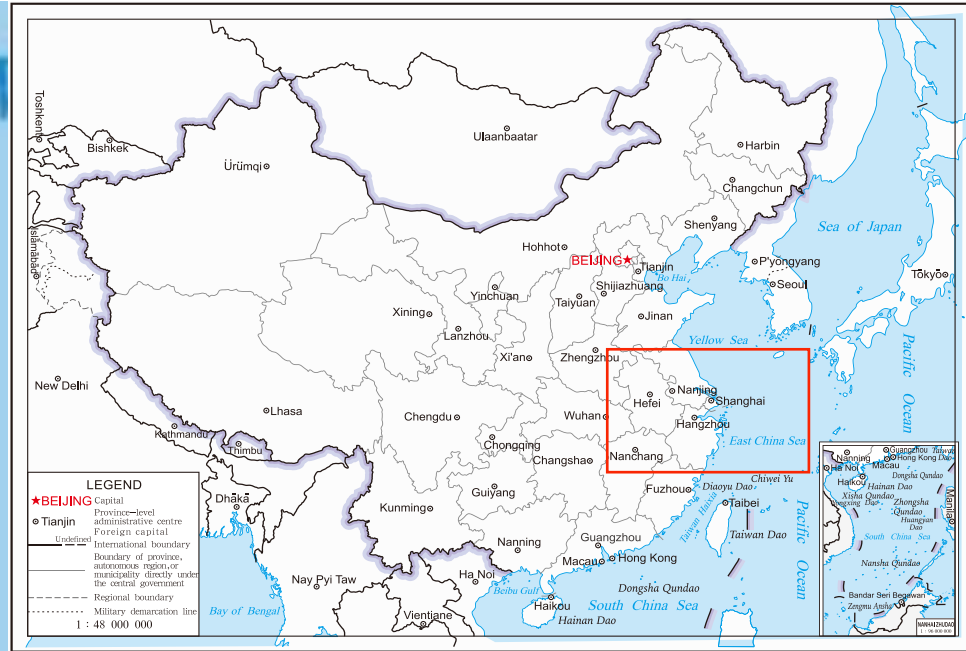

Approval number: GS(2019)1674

Supplement: Web_Material_uhae166 [file web_material_uhae166.zip › Figure S8. Geographical location of Ningbo (NB) and Nanjing (NJ) populationï1⁄4Othe approval number for the China map is GS(2019)1674.pdf]

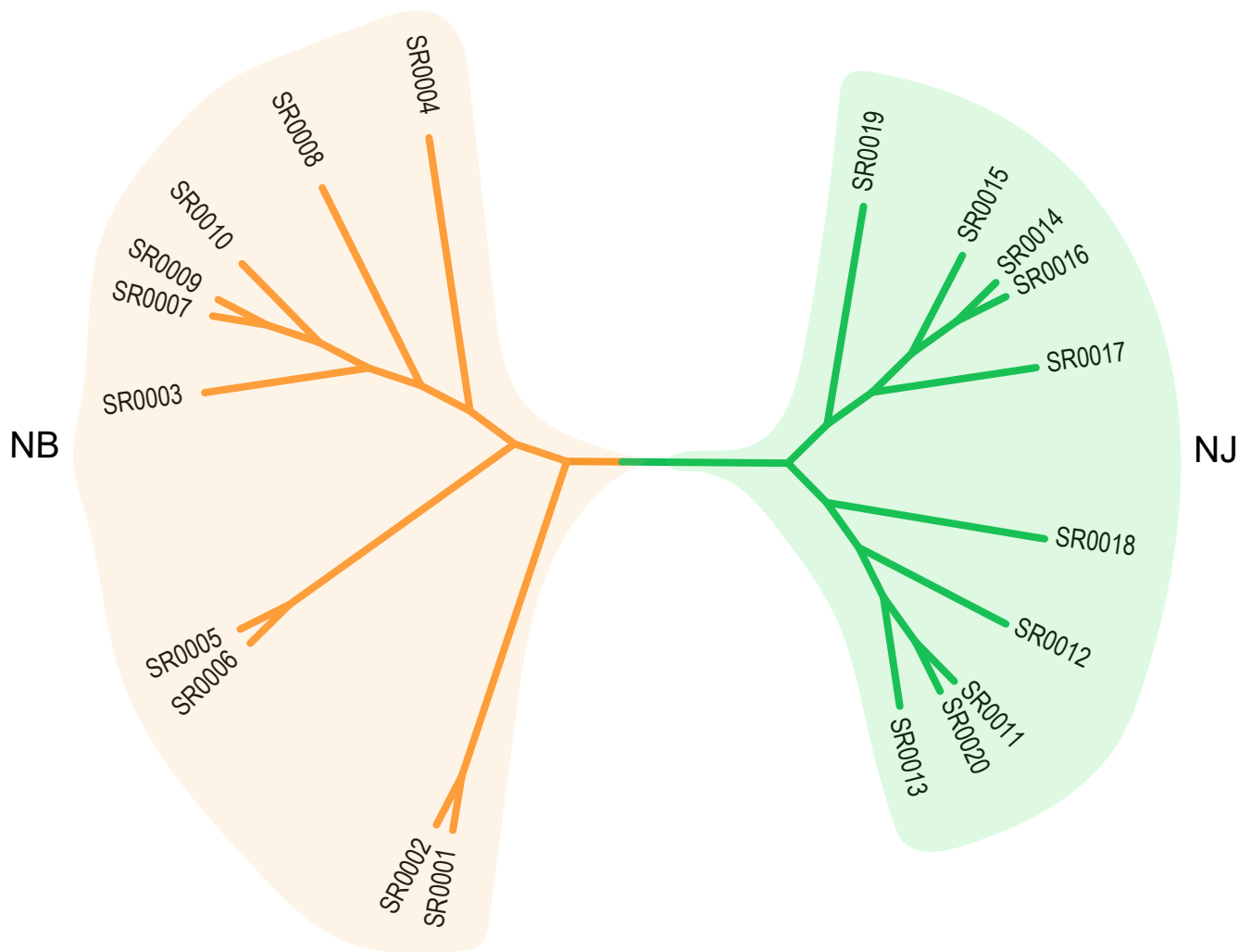

Supplement: Web_Material_uhae166 [file web_material_uhae166.zip › Figure S9. SNP-based phylogenetic tree of the 20 sequenced samples.pdf]
